# Supplementary material for: Protocol for the economic evaluation of COVID-19 pandemic response policies
Source: BMJ Open. 2021 Sep 14;11(9):e051503. doi: 10.1136/bmjopen-2021-051503 (PMC8441219; doi:10.1136/bmjopen-2021-051503)
Supplement: Supplementary data [file bmjopen-2021-051503supp001.pdf]

SUPPLEMENTARY APPENDIX: QUANTIFYING ECONOMIC COST OF NON-PHARMACEUTICAL INTERVENTIONS

Productivity loss

There are two types of productivity losses: COVID-19 infection related and wider population productivity loss. COVID-19 infection related productivity loss applies to those who are required to undergo isolation or quarantine because they are infected or are in close contacts to people with COVID-19 infection. The productivity loss for patients serving a compulsory quarantine order depends on the ability and productivity of working from home and the duration of the quarantine order. Wider population productivity loss occurs as a result of broad preventive NPIs such as workplace closure or workplace distancing as opposed to being directly affected by COVID-19. This productivity loss is attributable to the nature of jobs and services provided, level of infrastructure to support remote working, and individual companies' operations. Both categories of productivity loss must take into considerations the percentage of work that can be done from home. For example, Makridis et.al used digital-labour intensity for each industries in the US to estimate the proportion of work that can be done remotely.[1] Other countries such as Canada and Australia have made public servants work remotely even as the lockdown and social distancing eases.[2, 3] These policies for public servants can be used as a proxy to estimate the proportion of work that can be done remotely. Clearly, this is a variable that needs to be adapted to local settings.

In addition, productivity loss can be attributed to school closures. The United Nations Educational, Scientific and Cultural Organization has identified high economic costs among parents who miss work due to unavailable alternatives to childcare arrangements.[4] This could cause further strain the healthcare system if these parents are healthcare providers. Furthermore, there is large disparity in support for home learning between income levels.[5] This translate to loss of learning, especially among the disadvantaged minorities in the population. The United Nations has further identified the potential for rippled effects of school closures beyond learning with higher drop-out rates among poorer communities, which have direct economic consequences in potential future earnings for student affected.[6] This impact is particularly adverse during the foundation years of education, where a three-month school closure during grade 3 in developing countries is estimate to lead to higher drop-out rate by Grade 10,[7] and $16,000 income loss over a student's lifetime.[8]

Therefore, it is important to consider the opportunity costs due to productivity loss directly and indirectly attributable to COVID-19 and NPIs.

Test-trace-isolate policies costs:

Contract tracing activities will depend on the number of positive cases identified. The European Centre For Disease Control and Prevention guidance on contact tracing has suggested resource estimates of 2-3 and 7-20 close contacts during lockdown and pre-lockdown period, respectively.[9] Contact tracing manpower operational cost depends on the efficiency of contact tracing networks (how may contacts each contact tracing staff can process) and the mobility of infected individuals (social contacts) multiplied by tracers' wages and the necessary infrastructure cost to maintain the operation.

Travel restriction

To our knowledge, travel restriction has not been featured in any published cost-effectiveness analysis of vaccines given that border closures (between countries and between states/ provinces) of the current magnitude has been unprecedented. Global tourism and hospitality have experienced extreme decline.[10] Tourism and hospitality contribute a substantial portion to global economy in terms of

tourist receipts, employment, and infrastructure investments,[11] and is particularly critical for open economies such as Singapore. As such, border closure or travel restriction represents significant opportunity cost to countries. Opportunity cost of loss of tourism can be approximated from respective national economic databases. For example, in 2019, the Singapore tourism revenue reached SGD 27.7 billion (USD 20.4 billion), contributed 5.5% of the GDP and created 206,200 jobs.[12-14] In 2019, Vietnam tourism revenue reach USD 31 billion, contributed 6.3% of GDP, and created 4,951,000 jobs.[15] The difference of these revenue between 2020 and 2019 can be utilized as an estimation for the opportunity cost due to tourism. This tourism revenue represents the opportunity cost of NPIs, such as the potential benefit gain when travel is resumed upon the introduction of COVID-19 vaccines. This is likely a conservative estimation of opportunity costs as tourism receipts are likely to have larger ripple effect throughout the economies due to multiplier effect, especially for those relying heavily on tourism sector such as Thailand and Vietnam.

Compulsory face mask

The implementation of compulsory face mask policy is currently not universal. Hence, this cost parameter may be dropped if a jurisdiction does not impose compulsory use of face mask. The costs should reflect the economic costs of the masks and the resources required for monitoring and enforcing the policy. First, mandatory face masks will incur a societal cost borne by either the citizens or by governments (in the case where masks are freely distributed to the population). Second, manpower and administrative cost are incurred in monitoring and enforcement of the policy. However, there needs to be careful consideration of the assignment of cost. For example, in Singapore, Safe Distancing Ambassadors (SDA) are employed by the government to ensure public compliance with masks and social distancing policies.[16] The SDAs are usually individuals who have been displaced from their current jobs. Hence, it will be appropriate to include the cost of hiring these individuals. However, in some countries, the monitoring and enforcement may involve redeploying existing manpower and such cost may not need to be considered. For example, UK redeploys its police force to ensure mask compliance.[17]

Social distancing

There are many disadvantages associated with prolonged school closures. Hence, it is very likely that as COVID-19 lingers on, most countries will adopt as-needed school closure in response to clusters forming in schools rather than indefinite school closure. As a result of school closures, parents and guardians (working dependents) of the affected students would incur productivity loss related to childcare activities such as time loss from work or direct non-medical cost such as hiring of babysitters and part-time domestic helpers to help with household chores. This can be estimated by approximating forced absenteeism among working caregivers due to school closures and wages forgone.[18] Studies have shown that 12-13 week school closure in the UK during influenza pandemic in 2009 led to 0.2-1% GDP reduction.

The same considerations may be applied to as-needed workplace closure. As the COVID-19 SEIR model incorporates contact matrices to characterise the spread of infections, it is possible to estimate the number of individuals who will be affected with each school or workplace closure.

Currently, the impacts of WFH policies or remote working settings on productivity are not well characterised. As such, to estimate the economic impacts of WFH, we will vary the productivity penalty. In our base case analysis, we will assume that there is a productivity loss of 10%. Current evidence from working from home (or remote working) based on a Chinese travel agency reported a 13% increase in

productivity.[19] However, in a HRM Asia report, 20% of the interviewed workers in Singapore reported decline in productivity and 33% reported an increase in productivity.[20]

We will estimate the proportion of the workforce that can work from home, which is up to 80% in Singapore,[21] which will differ in other jurisdictions, especially in lower-and-middle-income countries. This data can be obtained from published government guidelines for essential services, or using the percentage of workforce in each industry as a proxy. For example, employees with deskbound duties can work from home, while those in the manufacturing sector who require hands-on operations cannot.

Social distancing and/or partial lockdown have caused substantial economic loss to businesses. This loss can be estimated from economic data (retail sales, consumers expenditure, etc.) or projected based on weighted impacts from various sectors, ranging from hardest-hit contact-intensive personal care industries (up to 90% business loss) to mildly affected low-contact industries.[22, 23] For example, Leibovici et al. found that in the US, 85% of the production of high contact-intensive industries are directly consumed (final demand) as compared to 39% for low contact-intensive industries. This means a decline in high contact-intensive industries will lead to a greater rippled effect in consumption via input-output linkages. Furthermore, we believed there are rippled effects to the economy as consumers behaviours are quickly adapting to prolonged limited social gathering.[24]

According to IFO Institute for Economic Research (IFO), an estimate production loss ratio can be applied to various economic activities under strict lockdown scenarios.[25] We can apply this ratio and weighted composition of services sector to derive total economic loss. The robustness test can be done by varying the ratio to reflect specific country's lockdown stringency. For example, "motion pictures, video and television programme production" may be deemed as non-essential services and thus production level ratio can be set to 0 (instead of 1 in IFO's scenario) for strict social lockdown. In Singapore, using the lockdown production ratio provided in the IFO's calculation and sectoral GDP contribution in 2019 for Singapore, we expected that for a 4-week national lockdown (ie. "circuit breaker"), average lockdown production is 0.42, representing a 58% fall in production output in the month under lockdown.

In economies with heavy reliance on household consumptions for economic growth such as the US where final consumption of goods and services is at 82.1% of GDP in 2016,[26] social distancing measures that ban or restrict retail services will likely have major economic consequences.

REFERENCES

1. The Cost of COVID-19: A Rough Estimate of the 2020 US GDP Impact. https://www.mercatus.org/system/files/makridis-cost-covid-19-mercatus-v1.pdf. Accessed 23 July 2020.
2. Today: Canada public servants will still work remotely even as offices reopen – minister. https://www.todayonline.com/world/canada-public-servants-will-still-work-remotely-even-offices-reopen-minister. Accessed 23 July 2020.
3. The Guardian: Australian federal public servants finally told they can work from home during coronavirus crisis. https://www.theguardian.com/world/2020/mar/29/australian-federal-public-servants-finally-told-they-can-work-from-home-during-coronavirus-crisis. Accessed 23 July 2020.
4. United Nations Educational, Scientific and Cultural Organization: Adverse consequences of school closures. https://en.unesco.org/covid19/educationresponse/consequences. Accessed 22 November 2020.
5. Pew Research Centre: Lower-income parents most concerned about their children falling behind amid COVID-19 school closures. https://www.pewresearch.org/fact-tank/2020/04/15/lower-income-parents-most-concerned-about-their-children-falling-behind-amid-covid-19-school-closures/. Accessed 22 November 2020.
6. United Nations: Education during COVID-19 and beyond. https://www.un.org/development/desa/dspd/wp-content/uploads/sites/22/2020/08/sg_policy_brief_covid-19_and_education_august_2020.pdf. Accessed 22 November 2020.
7. RISE: Modeling the Long-Run Learning Impact of the COVID-19 Learning Shock: Actions to (More Than) Mitigate Loss. https://riseprogramme.org/publications/modeling-long-run-learning-impact-covid-19-learning-shock-actions-more-mitigate-loss. Accessed 22 November 2020.
8. The World Bank: COVID-19 Could Lead to Permanent Loss in Learning and Trillions of Dollars in Lost Earnings. https://www.worldbank.org/en/news/press-release/2020/06/18/covid-19-could-lead-to-permanent-loss-in-learning-and-trillions-of-dollars-in-lost-earnings. Accessed 22 November 2020.
9. European Centre for Disease Prevention and Control: Contact tracing for COVID-19: current evidence, options for scale-up and an assessment of resources needed. https://www.ecdc.europa.eu/sites/default/files/documents/COVID-19-Contract-tracing-scale-up.pdf. Accessed 07 July 2020.
10. Pew Research Center: More than nine-in-ten people worldwide live in countries with travel restrictions amid COVID-19. https://www.pewresearch.org/fact-tank/2020/04/01/more-than-nine-in-ten-people-worldwide-live-in-countries-with-travel-restrictions-amid-covid-19/. Accessed 15 July 2020.
11. TripAdvisor Strategic Insights: Sizing Worldwide Tourism Spending (or "GTP") & TripAdvisor's Economic Impact. https://mk0tainsightsjao4bom.kinstacdn.com/wp-content/uploads/2018/09/Worldwide-Tourism-Economics-2017-compressed.pdf. Accessed 19 July 2020.
12. Singapore Tourism Board: Tourism Sector Performance Q4 2019. https://www.stb.gov.sg/content/dam/stb/documents/statistics-marketing-insights/Quarterly-Tourism-Performance-Report/STB%20Q4%202019%20FA%20v7.pdf. Accessed 03 August 2020.
13. Department of Statistics: Singapore Economy 2019. https://www.singstat.gov.sg/modules/infographics/economy. Accessed 03 August 2020.
14. World Travel & Tourism Council, Singapore. https://tool.wttc.org/. Accessed 03 August 2020.
15. Forbes Vietnam: Vietnam welcomes 18 millions international tourists this year. https://forbesvietnam.com.vn/tin-cap-nhat/nam-nay-viet-nam-don-18-trieu-luot-khach-quoc-te-8674.html. Accessed 03 August 2020.

16. Today: Hiring of safe distancing ambassadors, enforcement officers to continue after circuit breaker ends: MEWR. https://www.todayonline.com/singapore/hiring-safe-distancing-ambassadors-enforcement-officers-continue-after-circuit-breaker. Accessed 01 July 2020.
17. The Telegraph: 'Unrealistic and unfair' to expect police to enforce face coverings in shops, says Police Federation. https://www.telegraph.co.uk/global-health/science-and-disease/coronavirus-news-face-masks-transport-compulsory-cases-deaths/. Accessed 23 July 2020.
18. Viner RM, Russell SJ, Croker H, Packer J, Ward J, Stansfield C, et al. School closure and management practices during coronavirus outbreaks including COVID-19: a rapid systematic review. Lancet Child Adolesc Health. 2020.
19. Bloom N, Liang J, Roberts J, Ying ZJ. Does working from home work? Evidence from a Chinese experiment. Q J Econ. 2015;130:165-218.
20. HRM Asia: A third of Singaporeans feel more productive working from home. https://hrmasia.com/a-third-of-singaporeans-feel-more-productive-working-from-home/. Accessed 02 July 2020.
21. The Straits Times: At least 350,000 still commuting to work in Singapore after reduction in essential services. https://www.straitstimes.com/singapore/at-least-350000-still-commuting-to-work-in-singapore-after-reduction-in-essential-services. Accessed 02 July 2020.
22. Federal Reserve Bank of St.Louis: How the Impact of Social Distancing Ripples through the Economy. https://www.stlouisfed.org/on-the-economy/2020/april/impact-social-distancing-ripples-economy. Accessed 23 July 2020.
23. Statistic Canada: Impact of social distancing measures on businesses, by business characteristics. https://www150.statcan.gc.ca/t1/tbl1/en/tv.action?pid=3310023701. Accessed 21 July 2020.
24. McKinsey & Company: Consumer sentiment and behavior continue to reflect the uncertainty of the COVID-19 crisis. https://www.mckinsey.com/business-functions/marketing-and-sales/our-insights/a-global-view-of-how-consumer-behavior-is-changing-amid-covid-19#. Accessed 22 July 2020.
25. Mandel A, Veetil VP. The economic cost of covid lockdowns: An out-of-equilibrium analysis. Available at SSRN 3588421. 2020.
26. The World Bank: Final consumption expenditure (% of GDP). https://data.worldbank.org/indicator/NE.CON.TOTL.ZS. Accessed 03 August 2020.
