# Supplementary material for: Protocol for the economic evaluation of COVID-19 pandemic response policies
Source: BMJ Open. 2021 Sep 14;11(9):e051503. doi: 10.1136/bmjopen-2021-051503 (PMC8441219; doi:10.1136/bmjopen-2021-051503)
Supplement: Author's manuscript [file bmjopen-2021-051503.draft_revisions.pdf]

BMJ Open

BMJ Open is committed to open peer review. As part of this commitment we make the peer review history of every article we publish publicly available.

When an article is published we post the peer reviewers’ comments and the authors’ responses online. We also post the versions of the paper that were used during peer review. These are the versions that the peer review comments apply to.

The versions of the paper that follow are the versions that were submitted during the peer review process. They are not the versions of record or the final published versions. They should not be cited or distributed as the published version of this manuscript.

BMJ Open is an open access journal and the full, final, typeset and author-corrected version of record of the manuscript is available on our site with no access controls, subscription charges or pay-per-view fees (http://bmjopen.bmj.com).

If you have any questions on BMJ Open’s open peer review process please email info.bmjopen@bmj.com

BMJ Open

# Protocol for the Economic Evaluation of COVID-19 Pandemic Response Policies


| | |
|---|---|
| Journal: | *BMJ Open* |
| Manuscript ID | bmjopen-2021-051503 |
| Article Type: | Protocol |
| Date Submitted by the Author: | 21-Mar-2021 |



| | |
|---|---|
| Complete List of Authors: | Chua, Brandon; National University of Singapore, Saw Swee Hock School of Public Health<br>Huynh, Vinh Anh; National University of Singapore, Saw Swee Hock School of Public Health<br>Lou, Jing; National University of Singapore, Saw Swee Hock School of Public Health<br>Goh, Fang Ting; National University of Singapore, Saw Swee Hock School of Public Health<br>Clapham, Hannah; National University Singapore Saw Swee Hock School of Public Health<br>Teerawattananon, Yot; National University Singapore Saw Swee Hock School of Public Health; Health Intervention and Technology Assessment Program<br>Wee, Hwee Lin; National University of Singapore, Department of Pharmacy; National University of Singapore, Saw Swee Hock School of Public Health |



| | |
|---|---|
| Keywords: | COVID-19, HEALTH ECONOMICS, HEALTH SERVICES ADMINISTRATION & MANAGEMENT, Public health < INFECTIOUS DISEASES |


SCHOLARONE™
Manuscripts

*I, the Submitting Author has the right to grant and does grant on behalf of all authors of the Work (as defined in the below author licence), an exclusive licence and/or a non-exclusive licence for contributions from authors who are: i) UK Crown employees; ii) where BMJ has agreed a CC-BY licence shall apply, and/or iii) in accordance with the terms applicable for US Federal Government officers or employees acting as part of their official duties; on a worldwide, perpetual, irrevocable, royalty-free basis to BMJ Publishing Group Ltd ("BMJ") its licensees and where the relevant Journal is co-owned by BMJ to the co-owners of the Journal, to publish the Work in this journal and any other BMJ products and to exploit all rights, as set out in our licence.*

*The Submitting Author accepts and understands that any supply made under these terms is made by BMJ to the Submitting Author unless you are acting as an employee on behalf of your employer or a postgraduate student of an affiliated institution which is paying any applicable article publishing charge ("APC") for Open Access articles. Where the Submitting Author wishes to make the Work available on an Open Access basis (and intends to pay the relevant APC), the terms of reuse of such Open Access shall be governed by a Creative Commons licence – details of these licences and which Creative Commons licence will apply to this Work are set out in our licence referred to above.*

*Other than as permitted in any relevant BMJ Author's Self Archiving Policies, I confirm this Work has not been accepted for publication elsewhere, is not being considered for publication elsewhere and does not duplicate material already published. I confirm all authors consent to publication of this Work and authorise the granting of this licence.*

For peer review only

# Protocol for the Economic Evaluation of COVID-19 Pandemic Response Policies

Brandon Wen Bing Chua[1], Vinh Anh Huynh[1], Jing Lou[1], Fang Ting Goh,[1] Hannah Clapham[1], Yot Teerawattananon[1,2], Hwee Lin Wee[1,3]

corresponding author: Hwee Lin Wee; email: weehweelin@nus.edu.sg; Tel: +65-6516 4975

[1] Saw Swee Hock School of Public Health, National University of Singapore, Singapore
[2] Health Intervention and Technology Assessment Program, Thailand
[3] Department of Pharmacy, Faculty of Science, National University of Singapore, Singapore

Word count: 3321

For peer review only



For peer review only - http://bmjopen.bmj.com/site/about/guidelines.xhtml

**Abstract**

**Introduction**: Several treatment options are available for COVID-19 to date. However, the use of a combination of non-pharmaceutical interventions (NPIs) is necessary for jurisdictions to contain its spread. Although the implementation cost of NPIs may be low from the healthcare system perspective, it can be costly when considering the indirect costs from the societal perspective. COVID-19 vaccination campaigns have begun in several countries worldwide. Nonetheless, the quantity of vaccines available remain limited over the next one to two years. A tool for informing vaccine prioritization that considers both cost and effectiveness will be highly useful. This study aims to identify the most cost-effective combination of COVID-19 response policies, using Singapore as an example.

**Methods and analysis**: An age-stratified Susceptible-Exposed-Infectious-Recovered (SEIR) model will be used to generate the number of infections stratified by disease severity under different intervention scenarios. Polices of interest include test-trace-isolate, travel restriction, compulsory face mask and hygiene practices, social distancing, dexamethasone/remdesivir therapy and vaccination. The latest phase 3 trial results and the World Health Organization Target Product Profiles for COVID-19 vaccines will be used to model vaccine characteristics. A cost (expected resource utilization and productivity losses) and quality-adjusted life years (QALYs) will be attached to these outputs for a cost-utility analysis. The primary outcome measure will be the incremental cost effectiveness ratio (ICER) generated from the incremental cost of policy alternatives expressed as a ratio of the incremental benefits (QALYs gained). Efficacy of policy options will be gathered from literature review and from its observed impacts in Singapore. Cost data will be gathered from healthcare institutions, Ministry of Health and published data. Sensitivity analysis such as threshold analysis and scenario analysis will be conducted.

**Ethics and dissemination:** Ethics approval was not required for this study. The study findings will be disseminated through peer-reviewed journals.


review only



For peer review only - http://bmjopen.bmj.com/site/about/guidelines.xhtml

**Strengths and limitations of this study**

- Robust age-stratified SEIR model with an extensive contact matrix of 16 age groups, 2 sub-populations and 3 different location of interest.
- Various combination of non-pharmaceutical interventions and pharmaceutical drugs are analysed with vaccine distribution strategies.
- The proposed costing framework may be adapted for various jurisdictions to guide timely collection of essential variables to accelerate decision-making process

For peer review only



For peer review only - http://bmjopen.bmj.com/site/about/guidelines.xhtml

## INTRODUCTION

The Coronavirus disease 2019 (COVID-19) is a highly transmissible infectious disease caused by a novel severe acute respiratory syndrome coronavirus 2 (SARS-CoV-2). As of 16 March 2021, COVID-19 has affected more than 120 million people and resulted in over 2.6 million deaths worldwide.[1] According to the Asian Development Bank, up to $8.8 trillion in global economic loss is expected due to COVID-19, equivalent to approximately 10% of the world gross domestic product (GDP).[2]

We have now entered a very exciting time with at least three COVID-19 vaccines gaining market authorization. Vaccination has already begun in several countries such as the United States, United Kingdom, Russia, China, Israel and United Arab Emirates.[3] Nonetheless, the quantity of vaccines available remain limited over the next one to two years. A tool for informing vaccine prioritization that takes cost and effectiveness into account will be highly useful.

Mathematical and computational simulation models can support policy makers in decision making. A total of seven different models have been evaluated by the COVID-19 Multi-Model Comparison Collaboration, all of which describe disease transmission mechanistically to capture the effect of interventions on transmission and predict the impact of interventions.[4] The models project the number of infections by different severity, allowing for a variety of health-related and resource use results to be evaluated. By attaching costs and health utilities to the output of these models, we will be able to evaluate the cost-effectiveness of various policy scenarios. Given the scarcity of both healthcare and non-healthcare resources, techniques such as cost-effectiveness analysis provides a means of efficiently allocating scarce resources.

To facilitate efficient resource allocation, this study protocol aimed to describe a framework for conducting economic evaluation of pandemic response policies in the context of COVID-19. A new framework is required given that non-pharmaceutical interventions (NPIs) play a much more important role compared to other viral infections such as influenza virus or varicella-zoster virus. The earlier cost-effectiveness models for influenza vaccine or varicella-zoster vaccine did not consider NPIs in an elaborate manner. Many challenges exist with conducting an economic evaluation involving COVID-19 response strategies especially with the dearth of vaccine data. At the same time, such evaluations may not be feasible upon the release of vaccine candidates, as prompt decisions by policy makers may be required within a limited time frame. Hence, this paper will also serve to guide the timely collection of key data fields before the arrival of vaccines, especially in settings where expertise in economic evaluation is limited.

## METHODS AND ANALYSIS

This paper describes the protocol of an economic evaluation of COVID-19 response measures with examples from the Singapore healthcare sector and societal perspective. The healthcare sector perspective, recommended by the Agency for Care Effectiveness (ACE) in Singapore,[5] is necessary to evaluate the impact of managing COVID-19, especially in circumstances where there are capacity limitations. The societal perspective, although not recommended by ACE, has been recommended by the Second Panel on Cost-effectiveness in Health and Medicine,[6] and is essential for understanding the broader social and economic costs attributed to the management of COVID-19.

### Cost-effectiveness analysis

A cost-effectiveness analysis (CEA) provides a framework for comparing the incremental cost and incremental benefits of alternative policy options. To do so, the costs and benefits associated with each policy option must be estimated separately. A Markov cohort model is commonly used in CEA to



For peer review only - http://bmjopen.bmj.com/site/about/guidelines.xhtml

simulate data beyond the observed period and also to integrate data inputs from multiple sources.[7] However, a Markov cohort model will be less appropriate for an infectious disease where considerations of dynamic transmission is critical to ensure an accurate prediction of events. The indirect effects of averted infections are also not captured in Markov models, potentially undervaluing the effects of an intervention.[8] In the context of a vaccination program, this is significant as the risk of infection among both vaccinated and non-vaccinated susceptible people decreases with vaccination as the extent of virus circulating in the community is reduced.

Hence, in this paper, we propose to adopt a Susceptible-Exposed-Infectious-Recovered (SEIR) transmission model (to be elaborated in the next section) and to attach cost and quality-adjusted life years (QALYs) to the outputs from the SEIR model (number of infections by disease severity). The difference in costs between the reference case and the policy alternative is divided by the difference in QALYs between the reference case and the policy alternative to obtain an incremental cost effectiveness ratio (ICER). The ICER will then be interpreted according to the willingness-to-pay threshold of the jurisdiction of interest.

In Figure 1, we provide an illustration of the possible combinations of policy options that should be evaluated. This list is derived from discussions with infectious disease clinicians, policy makers, health technology assessment experts and COVID-19 disease modelers. As an example in the Singapore context, the use of test-trace-isolate (TTI), mask and hygiene, and treatments for COVID-19,[9] are central to all policy strategies as they are not expected to be removed in the presence of a COVID-19 vaccine, particularly when vaccine supply is limited and 100% efficacy of the vaccine cannot be assumed. However, this should be adapted to each jurisdiction's context. In the reference case, travel restriction and social are considered together with the three policies described above. In the presence of the vaccine, the extent of relaxation of policies can be explored. This can be the complete removal of travel restriction (option 4) or social distancing (option 3) or both (option 1). In addition, a partial relaxation of all policy options in the reference case can be explored in the presence of a vaccine (option 2).

The key vaccine characteristics to be considered in this study will be guided by the World Health Organization (WHO) Target Product Profile (TPP),[10] as well as emerging data on the Pfizer, Moderna and Oxford-Astra Zeneca vaccines.[11-13] This include the indication of use, contradiction, target population, efficacy, safety, contraindications, dose regime, duration of protection, route of administration and product stability. The vaccination distribution strategies to be considered is determined by vaccine availability and population size to be vaccinated. Given the global demand for COVID-19 vaccines, it is unlikely that sufficient supplies are available for an entire country for several months from the release of a commercially available vaccine. Furthermore, manufacturers may choose to supply vaccines only to those who are willing to pay high prices. Hence, varying levels of vaccine supply availability should be examined in scenario analyses (e.g. 100,000 units per month, 500,000 units per month and 1,000,000 units per month). In addition, varying vaccine efficacy and prioritization of who should receive the limited vaccines will be considered for each vaccine distribution strategy.

**Model description**

An age structured extended SEIR model will be programmed in R using a differential equation model to generate the number of infections in Singapore over time by age group and stratified by disease severity (asymptomatic, mild-moderate, severe, very severe). A schematic outline of the transmission model is depicted in Figure 2. The population will be split into two subpopulations: those residing in the foreign worker dormitories (FWD) and those residing outside of FWD. This allows for different COVID-19 policies between the two subpopulations as implemented in Singapore (e.g. more aggressive testing policy in FWD) as well as to capture the very different transmission in the two groups. Each subpopulation is



For peer review only - http://bmjopen.bmj.com/site/about/guidelines.xhtml

further stratified into 16 age groups of 5 years interval starting from age 0. This is to account for varying probabilities of getting infected and developing symptoms at different severity levels and vaccine effectiveness according to age groups. The effectiveness of vaccines will be modelled based on the following parameters with vaccine reducing the 1) probability of getting infected when in contact with an infectious individual; 2) probability of being symptomatic after being infected; 3) probability of progression to severe disease post infection; 4) Infectiousness of infected individuals.

The interaction between the 32 groups (16 in FWD and 16 outside FWD) is described by contact matrices. For example, a subpopulation with 16 age groups at a location of interest (school/ workplace/ community) will generate a $16 \times 32$ contact matrix. The matrix will describe the average number of contacts with others made by an individual in a location of interest. The overall contact matrix for a subpopulation is a linear combination of the three location-specific contact matrices, with the coefficient for each location-specific matrix ranging between 0 to 1. The implementation of NPIs, which reduces interaction between individuals could be modelled by adjusting the relevant contact matrix coefficient (e.g. school closure modelled as a drop in the school contact matrix coefficient).

With the contact matrices and the probabilities of getting infected and developing symptoms, we could estimate the transmission rate for each age group in each subpopulation, and in turn estimate the force of infection at each time. For parameters for which there is great uncertainty or that influence the outcomes greatly, we will conduct sensitivity analyses, and assess how these results impact the conclusions reached. The entire population of Singapore based on population norms in 2019 will be simulated over a period of 1 year.

**Healthcare utilization**

We assume that for the general population there is no active surveillance although there are pockets of individuals such as those in high risk occupations who may need to undergo active surveillance. In addition, we assume that patients with asymptomatic infections do not incur any healthcare resources until they develop symptoms. In Singapore, all symptomatic patients will be isolated within the hospital. Patients with mild to moderate symptoms will be managed in isolation wards. Patients with severe symptoms will require ventilator but not intensive care. Patients with very severe symptoms will require intensive care.

**COVID-19 response policies (pharmaceutical and NPI) and its impacts**

The COVID-19 response policies included in this study are detailed in Table 1 and their impacts will be simulated in the transmission model. Briefly, the policies include: 1) TTI; 2) imposing travel restriction (international and/or domestic travel); 3) compulsory mask and improved hygiene practices; 4) social distancing: including workplace closures, school closures and work from home policies; 5) Dexamethasone and/or remdesivir use in COVID-19 patients with severe and very severe symptoms; 6) COVID-19 vaccination. A systematic review will be conducted to obtain efficacy estimates for the policies against COVID-19. The estimated impact of each policy, in the form of reduction in contacts, COVID-19 case numbers, or disease severity, will be utilized for this study. Data from the Singapore context will be used as much as possible, failing which, data sources from developed Asian economies such as Hong Kong, Korea and Japan are considered. The efficacy and safety data from the latest COVID-19 vaccine trials will be used, [11-13] and supplemented with the WHO TPP.[10]



For peer review only - http://bmjopen.bmj.com/site/about/guidelines.xhtml

**Table 1 Key COVID-19 response policies and descriptions**

| **Policies** | **Sub-policies** | **Descriptions** |
|---|---|---|
| Test-trace-isolate policy | Community testing | A combination of active testing within the community to identify positive cases, establish the chain of transmission and isolate positive cases to prevent further spread. |
| | Isolation of positive cases (or suspected positive cases) | Policy to require mild/moderate positive patients to be isolated either in their homes or in care facilities. This includes policy requiring international travelers to observe self-isolation for a fixed period of time. Where extensive contact tracing is available, people who have come into close contacts with COVID-19 positive patients are also required to serve stay-at-home notice or quarantine orders. |
| | Contact tracing initiatives | Government or state's initiatives to identify close contacts of COVID-19 cases to prevent the spread. |
| Travel restriction | - | International travel restriction implemented by the government to ban, limit or restrict international travelers. |
| Compulsory face masks and improved hygiene practices | - | Requirement to wear face masks or facial coverings when in public spaces. |
| Social Distancing | As-needed workplaces and schools closures | Requirement to shut down workplaces or educational facilities when a cluster or a number of cases are detected, in order to test exposed individuals and disinfect the affected locations. |
| | Compulsory work-from-home (WFH) policies | This policy is complementary with social lockdown. Companies are to continue its operation with their staff working from home. This policy may remain in place even after social lockdown has been lifted. |
| | Physical distancing | Sets of policies requiring social gathering to maintain minimum distance (1 to 2 meters) between members when outside of their homes or residences. This policy is often accompanied by restricting service capacity of business, mainly retails, malls and personal services to prevent overcrowding. |



For peer review only

For peer review only - http://bmjopen.bmj.com/site/about/guidelines.xhtml

| | | |
|---|---|---|
| Dexamethasone therapy | - | Intravenous dexamethasone 6mg once daily for up to 10 days among patients with severe and very severe symptoms |
| Remdesivir therapy | - | Intravenous remdesivir 200mg on day 1, followed by 100mg once daily for up to 10 days among patients with severe and very severe symptoms |
| Vaccination | - | Vaccination against SARS-CoV-2 |

**Measurement and valuation of health benefits**

Health benefits will be measured using quality-adjusted life years (QALYs). This is derived by multiplying the number of life years lived with a health utility weight to adjust for health-related quality of life (HRQoL). Published health utilities among patients with COVID-19 will be used.[14-16] When data on impact of long COVID on HRQoL emerge,[17] these may also be incorporated into the model. As medical isolation has shown to have negative impacts on health-related quality of life due to depression and anxiety, we will apply a reduction of QALY on all patients who were isolated for COVID-19.[18] In addition, we will estimate QALY losses due to vaccine-related adverse events based on influenza vaccine for mild/injection site reactions, severe anaphylaxis and severe grade 3 reactions requiring hospitalization.[19]

**Measurement and valuation of cost**

The summary of the sources of cost data and valuations are shown in Table 2. All costs will be expressed in 2021 Singapore dollars (SG$) as we expect that a vaccine will arrive only in 2021. This may be tweaked according to local circumstances as some countries have begun mass vaccination in December 2020. The analysis will be done for a time horizon of 1 year, assuming that the efficacy of the vaccine only lasts for one year. Hence no discounting will be applied for this study. Alternative time horizons and discount rates may be explored in scenario analyses.

**Table 2 Key study components and recommended sources**

| | | Units | Remarks | Sources |
|---|---|---|---|---|
| **Medical cost** | **COVID-19 treatment cost** | | | |
| | Mild/Moderate | $/Case | Estimated | Hospital database, Ministry of Health |
| | Severe | $/Case | Estimated | Hospital database, Ministry of Health |
| | Very severe | $/Case | Estimated | Hospital database, Ministry of Health |
| | Death | $/Case | Estimated | Hospital database, Ministry of Health |
| | **Vaccine related cost** | | | |



For peer review only - http://bmjopen.bmj.com/site/about/guidelines.xhtml

| | | | | |
|---|---|---|---|---|
| | Cost to treat adverse side effect (mild) | $/Case | Estimated | Literature review, COVID-19 vaccine trial data (when available) |
| | Cost to treat adverse side effect (severe) | $/Case | Estimated | Literature review, COVID-19 vaccine trials (when available) |
| | Cost per dose | $/Dose | Estimated | Government purchase agreements, hospital and clinic charges |
| | Cost of vaccine delivery device | $/Dose | Estimated | Government purchase agreements, hospital and clinic charges |
| **Productivity Loss** | **Number of productive days loss** | | | |
| | Mild/Moderate | Days | Based on length of hospital stay | Hospital database, Ministry of Health |
| | Severe | Days | Based on length of hospital stay | Hospital database, Ministry of Health |
| | Very severe | Days | Based on length of hospital stay | Hospital database, Ministry of Health |
| | Death | Days | Based on length of hospital stay | Hospital database, Ministry of Health |
| | **Population parameters** | | | |
| | Average age of infection/populatio n | Years | Calculated | Hospital database, Ministry of Health |
| | Age of retirement | Years | Estimated | Ministry of Manpower/Labour |
| | Expected life years | Years | Estimated | Worldometer, World Health Organization |
| | Employed Persons | Persons | Estimated | Ministry of Manpower/Labour, National Economic Survey |
| | Population of working adults (resident) | Persons | If applicable | Ministry of Manpower/Labour, National Economic Survey |



For peer review only - http://bmjopen.bmj.com/site/about/guidelines.xhtml

| | | | | |
|---|---|---|---|---|
| | Average monthly wage | $/Month | Calculated | Ministry of Manpower/Labour, National Economic Survey |
| | Average annual wage | $/Year | Calculated | Ministry of Manpower/Labour, National Economic Survey |
| | Interest rate | % | Estimated | Literature Review |
| | Discount rate | % | Estimated | Literature Review |
| | Wage loss per death | $/Death | Calculated based on average wage, average age, retirement age, discount rate | - |
| **Community testing** | Cost per swab test | $/Test | Estimated | Hospital database, Ministry of Health |
| **Contact tracing** | Average monthly wage of contact tracers | $/Month | Estimated | Ministry of Health, Ministry of Manpower |
| | Number of Close Contacts traced per case | Contacts/Case | Estimated | Ministry of Health, European Centre For Disease Control and Prevention guidance on contact tracing |
| | Number of Close Contacts traced per Officer | Contacts/Officer | Estimated | Ministry of Health, European Centre For Disease Control and Prevention guidance on contact tracing |
| **Community isolation/quarantine** | Number of close contact per positive case | Persons | Estimated | Ministry of Health, European Centre For Disease Control and Prevention guidance on contact tracing |
| | Numbers of quarantined days for close cases | Days | Estimated | Ministry of Health, European Centre For Disease Control and Prevention guidance on contact tracing |



For peer review only - http://bmjopen.bmj.com/site/about/guidelines.xhtml

| | | | | |
|---|---|---|---|---|
| **Travel restriction** | Accommodation receipts loss | $/Year | Estimated | External research databases, National Tourism survey, Economic surveys tourism sector |
| | Food & beverages receipt loss | $/Year | Estimated | External research databases, National Tourism survey, Economic surveys tourism sector |
| | Shopping receipt loss | $/Year | Estimated | External research databases, National Tourism survey, Economic surveys tourism sector |
| | Sightseeing, entertainment & gaming receipt loss | $/Year | Estimated | External research databases, National Tourism survey, Economic surveys tourism sector |
| | Others receipt loss | $/Year | Estimated | External research databases, National Tourism survey, Economic surveys tourism sector |
| | Monthly visitor | Visitor/Month | Estimated | External research databases, National Tourism survey, Economic surveys tourism sector |
| | Average visitor's expenditure | $/Visitor | Total tourism receipt / tourists | Based on tourism receipts and number of tourist arrivals: Total tourism receipt / Number of tourist arrival |
| **Mask Cost** | Cost per mask | $/Item | Estimated | Marketplaces price, B2B platform prices with mark up for distribution (if applicable) |



For peer review only - http://bmjopen.bmj.com/site/about/guidelines.xhtml

| | | | | |
|---|---|---|---|---|
| | Cost of masks distributed to the public per month | $/Month | Calculated based on cost of mask, population and distribution mark-up factor | - |
| **Social distancing** | Number of social distancing officers | Person | Estimated | Ministry of Health |
| | Average monthly wage of Social Distancing Officers | $/Month | Estimated | Ministry of Health, Ministry of Manpower |
| **Cost due to as-needed School Closure** | Probability of one case in school resulting in school cluster/school closure | Probability | Estimated | Ministry of Health, Ministry of Education |
| | Number of children per school | Children/School | Estimated | Ministry of Education |
| | Duration of school closure | Months | Estimated | Expert opinion |
| | Productivity loss of working dependents | % | Estimated | Literature review on productivity loss, Human resource reports and Business Management studies |
| **Cost due to as-needed workplace closure** | Probability of one case in workplace resulting in workplace cluster/workplace closure | Probability | Estimated | Ministry of Health |
| | Number of people affected per closure | People/Case | Estimated | Ministry of Health, Ministry of Manpower |
| | Duration of workplace closure | Months | Estimated | Ministry of Health |
| **Work from home policy** | Productivity loss from WFH | % | Estimated | Literature review on productivity loss, Human resource reports and Business Management studies |



For peer review only - http://bmjopen.bmj.com/site/about/guidelines.xhtml

| | | | | |
|---|---|---|---|---|
| | Proportion of workers working from home | % | Estimated | Ministry of Health, Ministry of Manpower |
| **Impact on retails and services (Limited social gathering)** | Loss in income from Retail and Other services | $/Month | Estimated | National economic survey |
| | Loss in income from retail sales (under full Circuit Breaker measures) | $/Month | Estimated | National economic survey |
| | Loss income from food & beverage (under full Circuit Breaker measures) | $/Month | Estimated | National economic survey |
| | Income loss after reopening | % | Estimated | National economic survey |
| **Economic Loss from unemployment** | Total unemployment increase | Cases/Month | Estimated | Based on national economic survey, reports, international databases |
| | Average duration of unemployment | Months | Estimated | Literature Review based on unemployment and transitioning |
| | Total wage loss due to unemployment resulted over 12-month period | $ | Unemployment x Wage x Unemployment duration | Based on average wage and duration of unemployment Unemployment x Wage x Unemployment duration x time horizon |
| **Vaccines parameters** | Number of doses required | Doses | Assumed | WHO Target Product Profile, COVID-19 vaccine trials (when available) |
| | Efficacy | % | Estimated | WHO Target Product Profile, COVID-19 vaccine trials (when available) |
| | Proportion of population covered by vaccine | % | Estimated | Ministry of Health, WHO subnational vaccine coverage data |



For peer review only - http://bmjopen.bmj.com/site/about/guidelines.xhtml

| | | | | |
|---|---|---|---|---|
| | Mild adverse reaction (injection reaction and systemic adverse reactions) | % | Estimated | WHO vaccine reaction rates, COVID-19 vaccine trials (when available) |
| | Severe anaphylaxis | % | Estimated | WHO vaccine reaction rates, COVID-19 vaccine trials (when available) |
| | Grade 3 adverse reactions requiring hospitalisation | % | Estimated | WHO vaccine reaction rates, COVID-19 vaccine trials (when available) |
| **Health-utility** | **COVID-19 related** | | | |
| | Healthy | QALY | Estimated | Literature review on COVID-19 health utilities |
| | Mild/moderate symptoms | QALY | Estimated | Literature review on COVID-19 health utilities |
| | Severe symptoms | QALY | Estimated | Literature review on COVID-19 health utilities |
| | Very severe symptoms | QALY | Estimated | Literature review on COVID-19 health utilities |
| | Death | QALY | Assumed as 0 | - |
| | **Medical isolation** | | | |
| | Disutility due to medical isolation | QALY | Estimated | Literature review on medical isolation |
| | **Vaccine-related side effects** | | | |
| | Mild adverse reaction (injection reaction and systemic adverse reactions) | QALY | Estimated | Literature review on influenza vaccination |
| | Severe anaphylaxis | QALY | Estimated | Literature review on influenza vaccination |



For peer review only - http://bmjopen.bmj.com/site/about/guidelines.xhtml

| | | | | |
|---|---|---|---|---|
| | Grade 3 adverse reactions requiring hospitalisation | QALY | Estimated | Literature review on influenza vaccination |

Based on the healthcare system's perspective, direct medical costs will include costs to manage the infections stratified by severity, cost to manage adverse events associated with the vaccine and costs of the vaccination programme. Costs to manage the infections include hospitalization, medications, laboratory tests, diagnostic tests, radiological examinations, use of ventilators, medical procedures, etc, where applicable. The cost of COVID vaccine will be based on the acquisition price, cost of accompanying delivery device (if any) and the costs associated with cold-chain management when it becomes available but may otherwise be estimated based on reported spending of UK and US government on COVID-19 vaccine deals.[20, 21]

From the societal perspective, direct non-medical costs such as childcare services and indirect cost such as lost productivity will be included. There are two possible approaches to quantifying the economic cost of NPIs. One approach, which is being explored by our team, is to use stock market performance as a proxy. This is based on the assumption that the stock market performance is sensitive to any announcement of NPIs, e.g. stock market index falls following announcement of border closure due to concerns over the impact on businesses. The change in stock market indices may then be tied to GDP to quantify the economic losses in absolute dollar terms. The reason for not using GDP directly is because GDP data are reported on a quarterly basis and may not be as sensitive to announcements of NPI compared to stock market indices. This approach of using stock market index as a proxy has been used in other studies to measure investors' sentiment and expected economic activities, which in turn reflect investors' own perceptions of the economic impacts of NPIs.[22, 23] Another approach is more labour intensive and is elaborated in the Supplementary appendix.

**Sensitivity analyses**

Sensitivity analyses is a critical component of a cost-effectiveness analysis to examine the large number of assumptions made as well as the uncertainties associated with model parameters. Threshold analysis may be performed to determine the threshold cost of the vaccine under the various target product profiles modelled. Scenario analysis will consider different time horizons, vaccine characteristics and vaccine prioritization policies in this study.

Value of information analysis (VIA) could be applied to measure the gains from additional research which reduce the uncertainty in the model parameters. In particular, based on the results from sensitivity analysis, we could identify which parameters the ICER is most sensitive to. Those parameters could be considered for additional research to reduce uncertainties. Then we can decide whether to proceed with the additional research, by comparing the gains from the research (quantified in VIA) with the cost of conducting the research. If the additional research needed is prospective, VIA can also help to decide how much data to be further collected, thus optimizing the research design.

One limitation of using VIA here is that due to the urgency of COVID-19, new policies may need to be implemented immediately with the emergence of new clinical evidence. Hence there may not be sufficient time for additional prospective research before vaccine approval and distribution. However, VIA could help in at least two ways. Firstly, if VIA shows that reducing the uncertainty for selective parameters is of high value, it may be necessary to review if all existing information of those variables have been utilized. Secondly, VIA could inform the optimal post-licensure data collection needs for



For peer review only - http://bmjopen.bmj.com/site/about/guidelines.xhtml

monitoring the benefit-risk profile of vaccines, which includes any possible enhancement impact in post-licensure studies, and will help to determine future vaccination strategies beyond the initial doses.

## DISCUSSION

Several methodological challenges exist for conducting an economic evaluation of COVID-19 pandemic response strategies. Firstly, local data of the effectiveness of NPI may not be available for analysis. At the same time, effectiveness data of NPIs from another country setting are not directly applicable as they rely heavily on ecological factors, unlike pharmaceutical products that rely more on physiological and biological factors. Secondly, for vaccines, the long -term safety profile of COVID-19 vaccines will not be known, which may underestimate the risk associated with its use. In addition, uncertainties surrounding the long-term effectiveness of each vaccine candidate remains to be evaluated. Hence economic evaluation should be conducted once at the introduction of the vaccine with a subsequent analysis when more robust data is available. Thirdly, the population group to receive priority vaccination in the setting of limited vaccine supply remains to be determined. Available vaccine prioritization guidance by the WHO and Joint Committee on Vaccination and Immunisation have given health workers and the elderly the highest priority based on the goals of preserving health system capacity and minimizing COVID-related deaths.[24, 25] However, other jurisdictions may prioritize vaccination based on the goal of economic recovery, or a combination of both health-related and economic goals. These considerations would vary widely between jurisdictions, each with significant ethical considerations. Furthermore, other factors that may threaten vaccine uptake rates should be examined such as vaccine acceptance, payment models for vaccination, and expectations of daily living post-vaccination. Yet, efforts for economic evaluations of these COVID-19 pandemic response strategies should not be hampered as extensive sensitivity and scenario analysis of these known variables that contribute to large uncertainties can be conducted. Hence, a greater challenge would then lie in the interpretation of the results considering all these limitations.

As the COVID-19 pandemic continues, there are many trade-offs that need to be made among the various policy options. Economic evaluation can provide one source of input to inform this complex decision-making process. The protocol that we have described provides a detailed guide to estimate the broader economic costs of various NPIs that are being used, in line with other publications that have recommended the need to consider broader societal costs.[26, 27] Previous studies have so far established a cost inventory without specific guidelines or recommendations on how the costs should be estimated. Acemonglu et al presented optimal lockdown strategies in the US using trade-off between mortality and economy protection efforts without explicitly deriving the costs and associated calculations from the relevant components.[28] Kim et al proposed a cost inventory of policy responses for COVID-19 and highlighted the importance of the considering broader societal costs without guidance on how to compute the relevant costs.[29]

The costing framework that we have developed aims to fill such gaps and to provide a standard tool through which economic evaluations for COVID-19 vaccines can be based upon. This framework also allows for the flexible analysis of different policy combinations according to a country's needs. Hence, the extent of relaxation of individual COVID-19 policies to be explored in the presence of a vaccine. By allocating specific cost associated with specific policies, this framework allows comparison between different policy combinations but more importantly to compare the costs between different scenarios. This is key to policy evaluation where long-term planning is highly time-sensitive and policies should be allowed to have flexible implementation. In addition, this protocol would also be of relevant to settings with limited economic evaluation expertise, as data management plans can be drawn up to facilitate timely collection of these essential variables to help accelerate the decision-making process.



For peer review only - http://bmjopen.bmj.com/site/about/guidelines.xhtml

In conclusion, we have described a costing framework which will be useful to researchers and policy makers who are interested to evaluate the cost-effectiveness of various policy options. The costing framework is described in great details to allow researchers and policy makers to add on relevant cost parameters which we have not considered, or remove cost parameters that are not relevant or are not available in their own jurisdictions.

**Author contributions:** HLW and HC conceived the study; HLW, HC and YT contributed to the design of the study and procurement of funding. BCWB, VAH, JL, FTG drafted the manuscript and all authors have read and approved the final manuscript.

**Funding:** The study was support by the National Medical Research Council, Ministry of Health, Singapore (COVID19RF3-0057). The National University of Singapore is a member of the International Decision Support Initiative, which facilitates funding support for publication fees from the Bill and Melinda Gates Foundation (OPP1134345). The National Medical Research Council, Ministry of Heath, Singapore and Bill and Melinda Gates Foundation have no direct role in the design of the study and collection, analysis, and interpretation of data and in writing the manuscript.

**Competing interests:** The authors declare they have no competing interests.

**Patient and public involvement:** Patients and/or the public were not involved in the design, or conduct, or reporting, or dissemination plans of this research.

**Patient consent for publication:** Not required.

For peer review only



For peer review only - http://bmjopen.bmj.com/site/about/guidelines.xhtml

## REFERENCES


1. Worldometer. COVID-19 Coronvirus Pandemic. https://www.worldometers.info/coronavirus/. Accessed 16 March 2021.
2. Asian Development Bank. COVID-19 Economic Impact Could Reach $8.8 Trillion Globally - New ADB Report. https://www.adb.org/news/covid-19-economic-impact-could-reach-8-8-trillion-globally-new-adb-report. Accessed 04 June 2020.
3. Council on Foreign relations. A Guide to Global COVID-19 Vaccine Efforts https://www.cfr.org/backgrounder/guide-global-covid-19-vaccine-efforts. Accessed 16 March 2021.
4. Decide Health Decision Hub. Model Fitness-for-Purpose Assessment Report. https://decidehealth.world/en/news/model-fitness-purpose-assessment-report. Accessed 09 September 2020.
5. Agency of Care Effectiveness. Medical technologies evaluation methods and process guide. https://www.ace-hta.gov.sg/docs/default-source/process-methods/ace-methods-and-process-guide-for-medical-technologies-evaluation-(1-oct-2018).pdf. Accessed 20 March 2021.
6. Sanders GD, Neumann PJ, Basu A, Brock DW, Feeny D, Krahn M, et al. Recommendations for conduct, methodological practices, and reporting of cost-effectiveness analyses: second panel on cost-effectiveness in health and medicine. JAMA. 2016;316:1093-103.
7. Komorowski M, Raffa J. Markov models and cost effectiveness analysis: applications in medical research. Secondary analysis of electronic health records: Springer; 2016. p. 351-67.
8. Pitman R, Fisman D, Zaric GS, Postma M, Kretzschmar M, Edmunds J, et al. Dynamic transmission modeling: a report of the ISPOR-SMDM modeling good research practices task force-5. Value Health. 2012;15:828-34.
9. National Centre for Infectious Diseases. Interim treatment guidelines for COVID-19. https://www.ncid.sg/Documents/Interim%20Treatment%20Guidelines%20for%20COVID-19%20v4%20%2831%20Aug%202020%29-%20final.docx%20for%20upload.pdf. Accessed 16 December 2020.
10. World Health Organization. WHO Target Product Profiles for COVID-19 Vaccines. https://www.who.int/who-documents-detail/who-target-product-profiles-for-covid-19-vaccines. Accessed 04 June 2020.
11. Voysey M, Clemens SAC, Madhi SA, Weckx LY, Folegatti PM, Aley PK, et al. Safety and efficacy of the ChAdOx1 nCoV-19 vaccine (AZD1222) against SARS-CoV-2: an interim analysis of four randomised controlled trials in Brazil, South Africa, and the UK. Lancet. 2020.
12. Polack FP, Thomas SJ, Kitchin N, Absalon J, Gurtman A, Lockhart S, et al. Safety and efficacy of the BNT162b2 mRNA covid-19 vaccine. The New England journal of medicine. 2020.
13. Baden LR, El Sahly HM, Essink B, Kotloff K, Frey S, Novak R, et al. Efficacy and safety of the mRNA-1273 SARS-CoV-2 vaccine. New England Journal of Medicine. 2021;384:403-16.
14. Ping W, Zheng J, Niu X, Guo C, Zhang J, Yang H, et al. Evaluation of health-related quality of life using EQ-5D in China during the COVID-19 pandemic. PloS one. 2020;15:e0234850.
15. Garrigues E, Janvier P, Kherabi Y, Le Bot A, Hamon A, Gouze H, et al. Post-discharge persistent symptoms and health-related quality of life after hospitalization for COVID-19. J Infect. 2020.
16. Arab-Zozani M, Hashemi F, Safari H, Yousefi M, Ameri H. Health-Related Quality of Life and its Associated Factors in COVID-19 Patients. Osong Public Health Res Perspect. 2020;11:296-302.
17. Meeting the challenge of long COVID. Nat Med. 2020;26:1803.
18. Sharma A, Pillai DR, Lu M, Doolan C, Leal J, Kim J, et al. Impact of isolation precautions on quality of life: a meta-analysis. J Hosp Infect. 2020;105:35-42.
19. Prosser LA, Bridges CB, Uyeki TM, Hinrichsen VL, Meltzer MI, Molinari NA, et al. Health benefits, risks, and cost-effectiveness of influenza vaccination of children. Emerg Infect Dis. 2006;12:1548-58.




For peer review only - http://bmjopen.bmj.com/site/about/guidelines.xhtml

20. The Straits Times: Britain nears $870 million Sanofi-GlaxoSmithKline Covid-19 vaccine deal: Report. https://www.straitstimes.com/world/europe/britain-nears-870-million-sanofi-glaxosmithkline-covid-19-vaccine-deal-report. Accessed 09 July 2020.
21. The Straits Times: In brief US secures 300 million possible vaccine doses. https://www.straitstimes.com/world/in-brief-us-secures-300-million-possible-vaccine-doses. Accessed 09 July 2020.
22. Narayan PK, Phan DHB, Liu G. COVID-19 lockdowns, stimulus packages, travel bans, and stock returns. Finance Res Lett. 2020:101732.
23. Ashraf BN. Economic impact of government interventions during the COVID-19 pandemic: International evidence from financial markets. J Behav Exp Finance. 2020;27:100371.
24. World Health Organization. WHO SAGE Roadmap For Prioritizing Uses Of COVID-19 Vaccines In The Context Of Limited Supply. https://www.who.int/publications/m/item/who-sage-roadmap-for-prioritizing-uses-of-covid-19-vaccines-in-the-context-of-limited-supply. Accessed 19 November 2020.
25. Joint Committee on Vaccination and Immunisation: updated interim advice on priority groups for COVID-19 vaccination. https://www.gov.uk/government/publications/priority-groups-for-coronavirus-covid-19-vaccination-advice-from-the-jcvi-25-september-2020/jcvi-updated-interim-advice-on-priority-groups-for-covid-19-vaccination. Accessed 19 November 2020.
26. Appleby J. Will covid-19 vaccines be cost effective—and does it matter? BMJ. 2020;371:m4491.
27. Neumann P, Cohen JT, Kim DD, Ollendorf DA. Consideration Of Value-Based Pricing For Treatments And Vaccines Is Important, Even In The COVID-19 Pandemic: Study reviews alternative pricing strategies (cost-recovery models, monetary prizes, advanced market commitments) for COVID-19 drugs, vaccines, and diagnostics. Health Aff. 2020:10.1377/hlthaff. 2020.01548.
28. Acemoglu D, Chernozhukov V, Werning I, Whinston MD. Optimal targeted lockdowns in a multi-group SIR model. NBER Working Paper. 2020;27102.
29. Daeho Kim D, Neumann PJ. Analyzing the Cost Effectiveness of Policy Responses for COVID-19: The Importance of Capturing Social Consequences. Med Decis Making. 2020;40:251-3.

For peer review only

## Figures

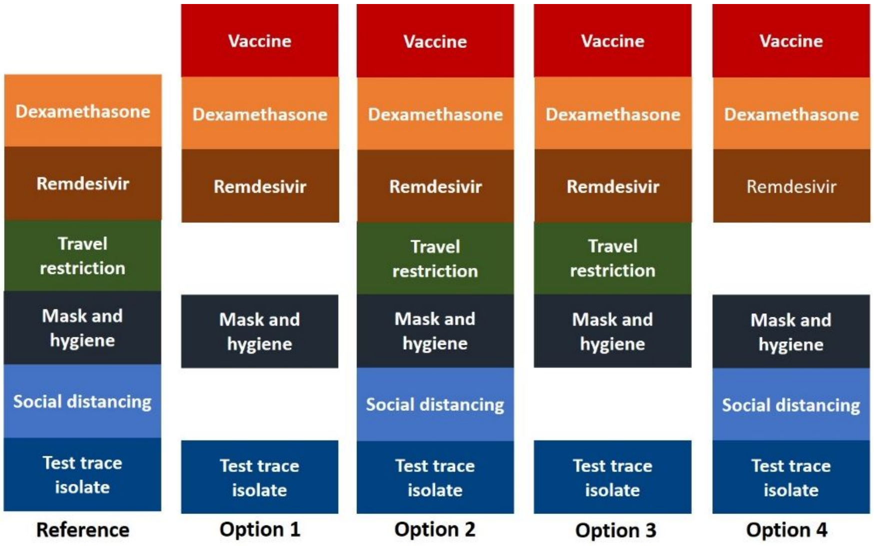


Figure 1. Combination of COVID-19 pandemic response policies

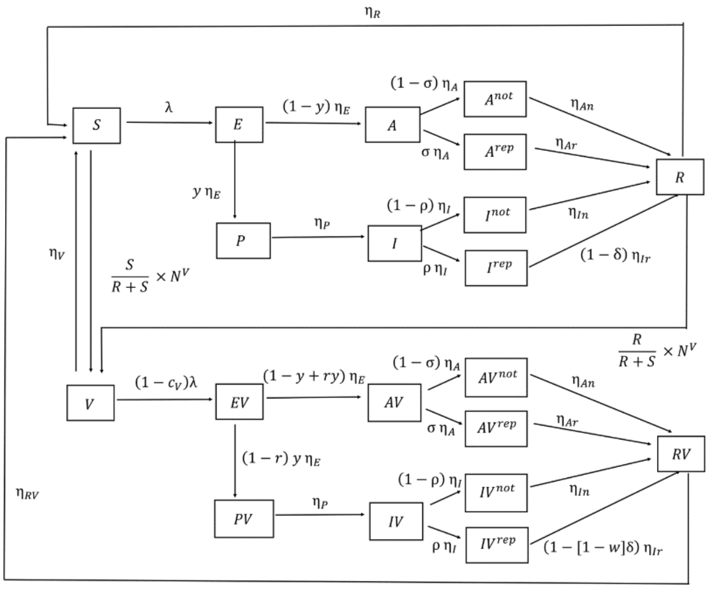

| **Compartments (Each compartment is split by age and by dorms and not dorms and each disease state have different infectivity)** |
|---|
| $S$ : Susceptible |
| $E$ : Exposed |
| $P$ : Presymptomatic |
| $I$ : Symptomatic |
| $A$ : Asymptomatic |
| $I^{rep}$ : Reported symptomatic |
| $I^{not}$ : Unreported symptomatic cases |
| $A^{rep}$ : Reported asymptomatic |
| $A^{not}$ : Unreported asymptomatic |
| $R$ : Recovered |
| $V$ : Vaccinated |
| $EV$ : Exposed after vaccination |
| $PV$ : Presymptomatic after vaccination |
| $IV$ : Symptomatic after vaccination |
| $AV$ : Asymptomatic after vaccination |
| $IV^{rep}$ : Reported symptomatic after vaccination |
| $IV^{not}$ : Unreported symptomatic after vaccination |
| $AV^{rep}$ : Reported asymptomatic after vaccination |
| $AV^{not}$ : Unreported asymptomatic cases after vaccination |
| $RV$ : Recovered after vaccination |

| **Parameters (May depend on age and on dorms and not dorms)** |
|---|
| $y$ : Probability of developing symptoms after getting infected |
| $\lambda$ : Force of infection (dependent on contact matrices and proportion of infected individuals) |
| $\sigma$ : Proportion of asymptomatic cases that are reported |
| $\rho$ : Proportion of symptomatic cases that are reported |
| $\delta$ : Proportion of deaths among reported symptomatic cases |
| $c_V$ : Effectiveness of vaccine in reducing probability of getting infected from an infectious individual |
| $r$ : Effectiveness of vaccine in reducing probability of being symptomatic after being infected |
| $w$ : Effectiveness of vaccine in reducing probability of death |
| $N^V$: Number of people who got vaccinated per unit time |
| $\eta_E$ : Incubation period |
| $\eta_P$ : Duration of presymptomatic state |
| $\eta_A$ : Time delay in reporting of asymptomatic cases |
| $\eta_I$ : Time delay in reporting of symptomatic cases |
| $\eta_{Ar}$ : Duration of stay in isolation facilities |
| $\eta_{An}$ : Recovery time for unreported asymptomatic cases after time delay in reporting |
| $\eta_{Ir}$ : Duration of stay in healthcare facilities until recovery |
| $\eta_{In}$ : Recovery time for unreported symptomatic cases after time delay in reporting |
| $\eta_V$ : Duration of vaccine protection |
| $\eta_R$ : Duration of immunity acquired from infection |
| $\eta_{RV}$ : Duration of immunity acquired from infection for vaccinated individuals |

Figure 2. Susceptible-Exposed-Infectious-Recovered transmission model schematics



For peer review only - http://bmjopen.bmj.com/site/about/guidelines.xhtml

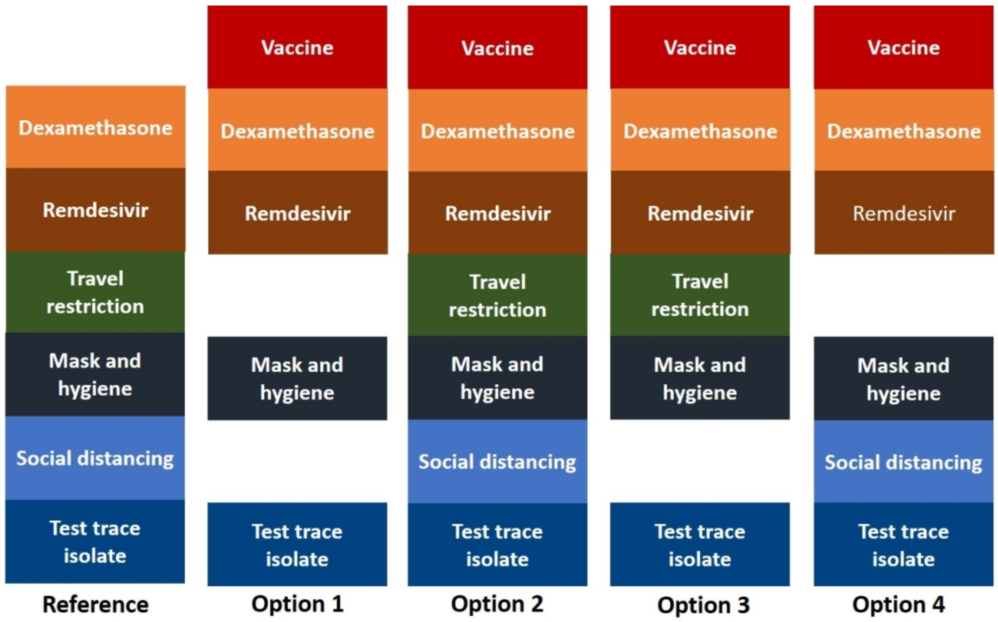


Figure 1. Combination of COVID-19 pandemic response policies

245x154mm (150 x 150 DPI)

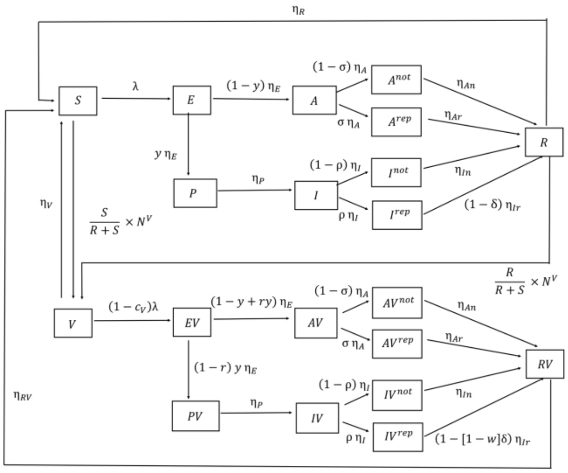


| **Compartments (Each compartment is split by age and by dorms and not dorms and each disease state have different infectivity)** |
|---|
| $S$ : Susceptible |
| $E$ : Exposed |
| $P$ : Presymptomatic |
| $I$ : Symptomatic |
| $A$ : Asymptomatic |
| $I^{rep}$ : Reported symptomatic |
| $I^{not}$ : Unreported symptomatic cases |
| $A^{rep}$ : Reported asymptomatic |
| $A^{not}$ : Unreported asymptomatic |
| $R$ : Recovered |
| $V$ : Vaccinated |
| $EV$ : Exposed after vaccination |
| $PV$ : Presymptomatic after vaccination |
| $IV$ : Symptomatic after vaccination |
| $AV$ : Asymptomatic after vaccination |
| $IV^{rep}$ : Reported symptomatic after vaccination |
| $IV^{not}$ : Unreported symptomatic after vaccination |
| $AV^{rep}$ : Reported asymptomatic after vaccination |
| $AV^{not}$ : Unreported asymptomatic cases after vaccination |
| $RV$ : Recovered after vaccination |

| **Parameters (May depend on age and on dorms and not dorms)** |
|---|
| $y$ : Probability of developing symptoms after getting infected |
| $\lambda$ : Force of infection (dependent on contact matrices and proportion of infected individuals) |
| $\sigma$ : Proportion of asymptomatic cases that are reported |
| $\rho$ : Proportion of symptomatic cases that are reported |
| $\delta$ : Proportion of deaths among reported symptomatic cases |
| $c_V$ : Effectiveness of vaccine in reducing probability of getting infected from an infectious individual |
| $r$ : Effectiveness of vaccine in reducing probability of being symptomatic after being infected |
| $w$ : Effectiveness of vaccine in reducing probability of death |
| $N^V$: Number of people who got vaccinated per unit time |
| $\eta_E$ : Incubation period |
| $\eta_P$ : Duration of presymptomatic state |
| $\eta_A$ : Time delay in reporting of asymptomatic cases |
| $\eta_I$ : Time delay in reporting of symptomatic cases |
| $\eta_{Ar}$ : Duration of stay in isolation facilities |
| $\eta_{An}$ : Recovery time for unreported asymptomatic cases after time delay in reporting |
| $\eta_{Ir}$ : Duration of stay in healthcare facilities until recovery |
| $\eta_{In}$ : Recovery time for unreported symptomatic cases after time delay in reporting |
| $\eta_V$ : Duration of vaccine protection |
| $\eta_R$ : Duration of immunity acquired from infection |
| $\eta_{RV}$ : Duration of immunity acquired from infection for vaccinated individuals |

Susceptible-Exposed-Infectious-Recovered transmission model schematics

## SUPPLEMENTARY APPENDIX: QUANTIFYING ECONOMIC COST OF NON-PHARMACEUTICAL INTERVENTIONS

### Productivity loss

There are two types of productivity losses: COVID-19 infection related and wider population productivity loss. COVID-19 infection related productivity loss applies to those who are required to undergo isolation or quarantine because they are infected or are in close contacts to people with COVID-19 infection. The productivity loss for patients serving a compulsory quarantine order depends on the ability and productivity of working from home and the duration of the quarantine order. Wider population productivity loss occurs as a result of broad preventive NPIs such as workplace closure or workplace distancing as opposed to being directly affected by COVID-19. This productivity loss is attributable to the nature of jobs and services provided, level of infrastructure to support remote working, and individual companies' operations. Both categories of productivity loss must take into considerations the percentage of work that can be done from home. For example, Makridis et.al used digital-labour intensity for each industries in the US to estimate the proportion of work that can be done remotely.[1] Other countries such as Canada and Australia have made public servants work remotely even as the lockdown and social distancing eases.[2, 3] These policies for public servants can be used as a proxy to estimate the proportion of work that can be done remotely. Clearly, this is a variable that needs to be adapted to local settings.

In addition, productivity loss can be attributed to school closures. The United Nations Educational, Scientific and Cultural Organization has identified high economic costs among parents who miss work due to unavailable alternatives to childcare arrangements.[4] This could cause further strain the healthcare system if these parents are healthcare providers. Furthermore, there is large disparity in support for home learning between income levels.[5] This translate to loss of learning, especially among the disadvantaged minorities in the population. The United Nations has further identified the potential for rippled effects of school closures beyond learning with higher drop-out rates among poorer communities, which have direct economic consequences in potential future earnings for student affected.[6] This impact is particularly adverse during the foundation years of education, where a three-month school closure during grade 3 in developing countries is estimate to lead to higher drop-out rate by Grade 10,[7] and $16,000 income loss over a student's lifetime.[8]

Therefore, it is important to consider the opportunity costs due to productivity loss directly and indirectly attributable to COVID-19 and NPIs.

### Test-trace-isolate policies costs:

Contract tracing activities will depend on the number of positive cases identified. The European Centre For Disease Control and Prevention guidance on contact tracing has suggested resource estimates of 2-3 and 7-20 close contacts during lockdown and pre-lockdown period, respectively.[9] Contact tracing manpower operational cost depends on the efficiency of contact tracing networks (how may contacts each contact tracing staff can process) and the mobility of infected individuals (social contacts) multiplied by tracers' wages and the necessary infrastructure cost to maintain the operation.

### Travel restriction

To our knowledge, travel restriction has not been featured in any published cost-effectiveness analysis of vaccines given that border closures (between countries and between states/ provinces) of the current magnitude has been unprecedented. Global tourism and hospitality have experienced extreme decline.[10] Tourism and hospitality contribute a substantial portion to global economy in terms of

For peer review only

tourist receipts, employment, and infrastructure investments,[11] and is particularly critical for open economies such as Singapore. As such, border closure or travel restriction represents significant opportunity cost to countries. Opportunity cost of loss of tourism can be approximated from respective national economic databases. For example, in 2019, the Singapore tourism revenue reached SGD 27.7 billion (USD 20.4 billion), contributed 5.5% of the GDP and created 206,200 jobs.[12-14] In 2019, Vietnam tourism revenue reach USD 31 billion, contributed 6.3% of GDP, and created 4,951,000 jobs.[15] The difference of these revenue between 2020 and 2019 can be utilized as an estimation for the opportunity cost due to tourism. This tourism revenue represents the opportunity cost of NPIs, such as the potential benefit gain when travel is resumed upon the introduction of COVID-19 vaccines. This is likely a conservative estimation of opportunity costs as tourism receipts are likely to have larger ripple effect throughout the economies due to multiplier effect, especially for those relying heavily on tourism sector such as Thailand and Vietnam.

**Compulsory face mask**

The implementation of compulsory face mask policy is currently not universal. Hence, this cost parameter may be dropped if a jurisdiction does not impose compulsory use of face mask. The costs should reflect the economic costs of the masks and the resources required for monitoring and enforcing the policy. First, mandatory face masks will incur a societal cost borne by either the citizens or by governments (in the case where masks are freely distributed to the population). Second, manpower and administrative cost are incurred in monitoring and enforcement of the policy. However, there needs to be careful consideration of the assignment of cost. For example, in Singapore, Safe Distancing Ambassadors (SDA) are employed by the government to ensure public compliance with masks and social distancing policies.[16] The SDAs are usually individuals who have been displaced from their current jobs. Hence, it will be appropriate to include the cost of hiring these individuals. However, in some countries, the monitoring and enforcement may involve redeploying existing manpower and such cost may not need to be considered. For example, UK redeploys its police force to ensure mask compliance.[17]

**Social distancing**

There are many disadvantages associated with prolonged school closures. Hence, it is very likely that as COVID-19 lingers on, most countries will adopt as-needed school closure in response to clusters forming in schools rather than indefinite school closure. As a result of school closures, parents and guardians (working dependents) of the affected students would incur productivity loss related to childcare activities such as time loss from work or direct non-medical cost such as hiring of babysitters and part-time domestic helpers to help with household chores. This can be estimated by approximating forced absenteeism among working caregivers due to school closures and wages forgone.[18] Studies have shown that 12-13 week school closure in the UK during influenza pandemic in 2009 led to 0.2-1% GDP reduction.

The same considerations may be applied to as-needed workplace closure. As the COVID-19 SEIR model incorporates contact matrices to characterise the spread of infections, it is possible to estimate the number of individuals who will be affected with each school or workplace closure.

Currently, the impacts of WFH policies or remote working settings on productivity are not well characterised. As such, to estimate the economic impacts of WFH, we will vary the productivity penalty. In our base case analysis, we will assume that there is a productivity loss of 10%. Current evidence from working from home (or remote working) based on a Chinese travel agency reported a 13% increase in

For peer review only

productivity.[19] However, in a HRM Asia report, 20% of the interviewed workers in Singapore reported decline in productivity and 33% reported an increase in productivity.[20]

We will estimate the proportion of the workforce that can work from home, which is up to 80% in Singapore,[21] which will differ in other jurisdictions, especially in lower-and-middle-income countries. This data can be obtained from published government guidelines for essential services, or using the percentage of workforce in each industry as a proxy. For example, employees with deskbound duties can work from home, while those in the manufacturing sector who require hands-on operations cannot.

Social distancing and/or partial lockdown have caused substantial economic loss to businesses. This loss can be estimated from economic data (retail sales, consumers expenditure, etc.) or projected based on weighted impacts from various sectors, ranging from hardest-hit contact-intensive personal care industries (up to 90% business loss) to mildly affected low-contact industries.[22, 23] For example, Leibovici et al. found that in the US, 85% of the production of high contact-intensive industries are directly consumed (final demand) as compared to 39% for low contact-intensive industries. This means a decline in high contact-intensive industries will lead to a greater rippled effect in consumption via input-output linkages. Furthermore, we believed there are rippled effects to the economy as consumers behaviours are quickly adapting to prolonged limited social gathering.[24]

According to IFO Institute for Economic Research (IFO), an estimate production loss ratio can be applied to various economic activities under strict lockdown scenarios.[25] We can apply this ratio and weighted composition of services sector to derive total economic loss. The robustness test can be done by varying the ratio to reflect specific country's lockdown stringency. For example, "motion pictures, video and television programme production" may be deemed as non-essential services and thus production level ratio can be set to 0 (instead of 1 in IFO's scenario) for strict social lockdown. In Singapore, using the lockdown production ratio provided in the IFO's calculation and sectoral GDP contribution in 2019 for Singapore, we expected that for a 4-week national lockdown (ie. "circuit breaker"), average lockdown production is 0.42, representing a 58% fall in production output in the month under lockdown.

In economies with heavy reliance on household consumptions for economic growth such as the US where final consumption of goods and services is at 82.1% of GDP in 2016,[26] social distancing measures that ban or restrict retail services will likely have major economic consequences.

For peer review only

**REFERENCES**


1. The Cost of COVID-19: A Rough Estimate of the 2020 US GDP Impact. https://www.mercatus.org/system/files/makridis-cost-covid-19-mercatus-v1.pdf. Accessed 23 July 2020.
2. Today: Canada public servants will still work remotely even as offices reopen – minister. https://www.todayonline.com/world/canada-public-servants-will-still-work-remotely-even-offices-reopen-minister. Accessed 23 July 2020.
3. The Guardian: Australian federal public servants finally told they can work from home during coronavirus crisis. https://www.theguardian.com/world/2020/mar/29/australian-federal-public-servants-finally-told-they-can-work-from-home-during-coronavirus-crisis. Accessed 23 July 2020.
4. United Nations Educational, Scientific and Cultural Organization: Adverse consequences of school closures. https://en.unesco.org/covid19/educationresponse/consequences. Accessed 22 November 2020.
5. Pew Research Centre: Lower-income parents most concerned about their children falling behind amid COVID-19 school closures. https://www.pewresearch.org/fact-tank/2020/04/15/lower-income-parents-most-concerned-about-their-children-falling-behind-amid-covid-19-school-closures/. Accessed 22 November 2020.
6. United Nations: Education during COVID-19 and beyond. https://www.un.org/development/desa/dspd/wp-content/uploads/sites/22/2020/08/sg_policy_brief_covid-19_and_education_august_2020.pdf. Accessed 22 November 2020.
7. RISE: Modeling the Long-Run Learning Impact of the COVID-19 Learning Shock: Actions to (More Than) Mitigate Loss. https://riseprogramme.org/publications/modeling-long-run-learning-impact-covid-19-learning-shock-actions-more-mitigate-loss. Accessed 22 November 2020.
8. The World Bank: COVID-19 Could Lead to Permanent Loss in Learning and Trillions of Dollars in Lost Earnings. https://www.worldbank.org/en/news/press-release/2020/06/18/covid-19-could-lead-to-permanent-loss-in-learning-and-trillions-of-dollars-in-lost-earnings. Accessed 22 November 2020.
9. European Centre for Disease Prevention and Control: Contact tracing for COVID-19: current evidence, options for scale-up and an assessment of resources needed. https://www.ecdc.europa.eu/sites/default/files/documents/COVID-19-Contract-tracing-scale-up.pdf. Accessed 07 July 2020.
10. Pew Research Center: More than nine-in-ten people worldwide live in countries with travel restrictions amid COVID-19. https://www.pewresearch.org/fact-tank/2020/04/01/more-than-nine-in-ten-people-worldwide-live-in-countries-with-travel-restrictions-amid-covid-19/. Accessed 15 July 2020.
11. TripAdvisor Strategic Insights: Sizing Worldwide Tourism Spending (or "GTP") & TripAdvisor's Economic Impact. https://mk0tainsightsjao4bom.kinstacdn.com/wp-content/uploads/2018/09/Worldwide-Tourism-Economics-2017-compressed.pdf. Accessed 19 July 2020.
12. Singapore Tourism Board: Tourism Sector Performance Q4 2019. https://www.stb.gov.sg/content/dam/stb/documents/statistics-marketing-insights/Quarterly-Tourism-Performance-Report/STB%20Q4%202019%20FA%20v7.pdf. Accessed 03 August 2020.
13. Department of Statistics: Singapore Economy 2019. https://www.singstat.gov.sg/modules/infographics/economy. Accessed 03 August 2020.
14. World Travel & Tourism Council, Singapore. https://tool.wttc.org/. Accessed 03 August 2020.
15. Forbes Vietnam: Vietnam welcomes 18 millions international tourists this year. https://forbesvietnam.com.vn/tin-cap-nhat/nam-nay-viet-nam-don-18-trieu-luot-khach-quoc-te-8674.html. Accessed 03 August 2020.


For peer review only

16. Today: Hiring of safe distancing ambassadors, enforcement officers to continue after circuit breaker ends: MEWR. https://www.todayonline.com/singapore/hiring-safe-distancing-ambassadors-enforcement-officers-continue-after-circuit-breaker. Accessed 01 July 2020.
17. The Telegraph: 'Unrealistic and unfair' to expect police to enforce face coverings in shops, says Police Federation. https://www.telegraph.co.uk/global-health/science-and-disease/coronavirus-news-face-masks-transport-compulsory-cases-deaths/. Accessed 23 July 2020.
18. Viner RM, Russell SJ, Croker H, Packer J, Ward J, Stansfield C, et al. School closure and management practices during coronavirus outbreaks including COVID-19: a rapid systematic review. Lancet Child Adolesc Health. 2020.
19. Bloom N, Liang J, Roberts J, Ying ZJ. Does working from home work? Evidence from a Chinese experiment. Q J Econ. 2015;130:165-218.
20. HRM Asia: A third of Singaporeans feel more productive working from home. https://hrmasia.com/a-third-of-singaporeans-feel-more-productive-working-from-home/. Accessed 02 July 2020.
21. The Straits Times: At least 350,000 still commuting to work in Singapore after reduction in essential services. https://www.straitstimes.com/singapore/at-least-350000-still-commuting-to-work-in-singapore-after-reduction-in-essential-services. Accessed 02 July 2020.
22. Federal Reserve Bank of St.Louis: How the Impact of Social Distancing Ripples through the Economy. https://www.stlouisfed.org/on-the-economy/2020/april/impact-social-distancing-ripples-economy. Accessed 23 July 2020.
23. Statistic Canada: Impact of social distancing measures on businesses, by business characteristics. https://www150.statcan.gc.ca/t1/tbl1/en/tv.action?pid=3310023701. Accessed 21 July 2020.
24. McKinsey & Company: Consumer sentiment and behavior continue to reflect the uncertainty of the COVID-19 crisis. https://www.mckinsey.com/business-functions/marketing-and-sales/our-insights/a-global-view-of-how-consumer-behavior-is-changing-amid-covid-19#. Accessed 22 July 2020.
25. Mandel A, Veetil VP. The economic cost of covid lockdowns: An out-of-equilibrium analysis. Available at SSRN 3588421. 2020.
26. The World Bank: Final consumption expenditure (% of GDP). https://data.worldbank.org/indicator/NE.CON.TOTL.ZS. Accessed 03 August 2020.

For peer review only

BMJ Open

# Protocol for the Economic Evaluation of COVID-19 Pandemic Response Policies

| | |
|---|---|
| Journal: | *BMJ Open* |
| Manuscript ID | bmjopen-2021-051503.R1 |
| Article Type: | Protocol |
| Date Submitted by the Author: | 24-Aug-2021 |
| Complete List of Authors: | Chua, Brandon; National University Singapore Saw Swee Hock School of Public Health<br>Huynh, Vinh Anh; National University of Singapore, Saw Swee Hock School of Public Health<br>Lou, Jing; National University of Singapore, Saw Swee Hock School of Public Health<br>Goh, Fang Ting; National University of Singapore, Saw Swee Hock School of Public Health<br>Clapham, Hannah; National University Singapore Saw Swee Hock School of Public Health<br>Teerawattananon, Yot; National University Singapore Saw Swee Hock School of Public Health; Health Intervention and Technology Assessment Program<br>Wee, Hwee Lin; National University of Singapore, Department of Pharmacy; National University of Singapore, Saw Swee Hock School of Public Health |
| <b>Primary Subject Heading</b>: | Health economics |
| Secondary Subject Heading: | Infectious diseases, Health policy, Public health |
| Keywords: | COVID-19, HEALTH ECONOMICS, HEALTH SERVICES ADMINISTRATION & MANAGEMENT, Public health < INFECTIOUS DISEASES |
| | |

SCHOLARONE™
Manuscripts

*I, the Submitting Author has the right to grant and does grant on behalf of all authors of the Work (as defined in the below author licence), an exclusive licence and/or a non-exclusive licence for contributions from authors who are: i) UK Crown employees; ii) where BMJ has agreed a CC-BY licence shall apply, and/or iii) in accordance with the terms applicable for US Federal Government officers or employees acting as part of their official duties; on a worldwide, perpetual, irrevocable, royalty-free basis to BMJ Publishing Group Ltd ("BMJ") its licensees and where the relevant Journal is co-owned by BMJ to the co-owners of the Journal, to publish the Work in this journal and any other BMJ products and to exploit all rights, as set out in our licence.*

*The Submitting Author accepts and understands that any supply made under these terms is made by BMJ to the Submitting Author unless you are acting as an employee on behalf of your employer or a postgraduate student of an affiliated institution which is paying any applicable article publishing charge ("APC") for Open Access articles. Where the Submitting Author wishes to make the Work available on an Open Access basis (and intends to pay the relevant APC), the terms of reuse of such Open Access shall be governed by a Creative Commons licence – details of these licences and which Creative Commons licence will apply to this Work are set out in our licence referred to above.*

*Other than as permitted in any relevant BMJ Author's Self Archiving Policies, I confirm this Work has not been accepted for publication elsewhere, is not being considered for publication elsewhere and does not duplicate material already published. I confirm all authors consent to publication of this Work and authorise the granting of this licence.*

For peer review only

# Protocol for the Economic Evaluation of COVID-19 Pandemic Response Policies

Brandon Wen Bing Chua[1], Vinh Anh Huynh[1], Jing Lou[1], Fang Ting Goh,[1] Hannah Clapham[1], Yot Teerawattananon[1,2], Hwee Lin Wee[1,3]

corresponding author: Hwee Lin Wee; email: weehweelin@nus.edu.sg; Tel: +65-6516 4975

[1] Saw Swee Hock School of Public Health, National University of Singapore, Singapore
[2] Health Intervention and Technology Assessment Program, Thailand
[3] Department of Pharmacy, Faculty of Science, National University of Singapore, Singapore

Word count: 3432

For peer review only

**Abstract**

**Introduction**: Several treatment options are available for COVID-19 to date. However, the use of a combination of non-pharmaceutical interventions (NPIs) is necessary for jurisdictions to contain its spread. Although the implementation cost of NPIs may be low from the healthcare system perspective, it can be costly when considering the indirect costs from the societal perspective. COVID-19 vaccination campaigns have begun in several countries worldwide. Nonetheless, the quantity of vaccines available remain limited over the next one to two years. A tool for informing vaccine prioritization that considers both cost and effectiveness will be highly useful. This study aims to identify the most cost-effective combination of COVID-19 response policies, using Singapore as an example.

**Methods and analysis**: An age-stratified Susceptible-Exposed-Infectious-Recovered (SEIR) model will be used to generate the number of infections stratified by disease severity under different intervention scenarios. Polices of interest include test-trace-isolate, travel restriction, compulsory face mask and hygiene practices, social distancing, dexamethasone/remdesivir therapy and vaccination. The latest phase 3 trial results and the World Health Organization Target Product Profiles for COVID-19 vaccines will be used to model vaccine characteristics. A cost (expected resource utilization and productivity losses) and quality-adjusted life years (QALYs) will be attached to these outputs for a cost-utility analysis. The primary outcome measure will be the incremental cost effectiveness ratio (ICER) generated from the incremental cost of policy alternatives expressed as a ratio of the incremental benefits (QALYs gained). Efficacy of policy options will be gathered from literature review and from its observed impacts in Singapore. Cost data will be gathered from healthcare institutions, Ministry of Health and published data. Sensitivity analysis such as threshold analysis and scenario analysis will be conducted.

**Ethics and dissemination:** Ethics approval was not required for this study. The study findings will be disseminated through peer-reviewed journals.

review only



For peer review only - http://bmjopen.bmj.com/site/about/guidelines.xhtml

**Strengths and limitations of this study**

- Robust age-stratified SEIR model with an extensive contact matrix of 16 age groups, 2 sub-populations and 3 different location of interest.
- Various combination of non-pharmaceutical interventions and pharmaceutical drugs are analysed with vaccine distribution strategies.
- The proposed costing framework may be adapted for various jurisdictions to guide timely collection of essential variables to accelerate decision-making process

For peer review only



For peer review only - http://bmjopen.bmj.com/site/about/guidelines.xhtml

## INTRODUCTION

The Coronavirus disease 2019 (COVID-19) is a highly transmissible infectious disease caused by a novel severe acute respiratory syndrome coronavirus 2 (SARS-CoV-2). As of 16 March 2021, COVID-19 has affected more than 120 million people and resulted in over 2.6 million deaths worldwide.[1] According to the Asian Development Bank, up to $8.8 trillion in global economic loss is expected due to COVID-19, equivalent to approximately 10% of the world gross domestic product (GDP).[2]

We have now entered a very exciting time with at least three COVID-19 vaccines gaining market authorization. Vaccination has already begun in several countries such as the United States, United Kingdom, Russia, China, Israel and United Arab Emirates.[3] Nonetheless, the quantity of vaccines available remain limited over the next one to two years. A tool for informing vaccine prioritization that takes cost and effectiveness into account will be highly useful.

Mathematical and computational simulation models can support policy makers in decision making. A total of seven different models have been evaluated by the COVID-19 Multi-Model Comparison Collaboration, all of which describe disease transmission mechanistically to capture the effect of interventions on transmission and predict the impact of interventions.[4] The models project the number of infections by different severity, allowing for a variety of health-related and resource use results to be evaluated. By attaching costs and health utilities to the output of these models, we will be able to evaluate the cost-effectiveness of various policy scenarios. Given the scarcity of both healthcare and non-healthcare resources, techniques such as cost-effectiveness analysis provides a means of efficiently allocating scarce resources.

To facilitate efficient resource allocation, this study protocol aimed to describe a framework for conducting economic evaluation of pandemic response policies in the context of COVID-19. A new framework is required given that non-pharmaceutical interventions (NPIs) play a much more important role compared to other viral infections such as influenza virus or varicella-zoster virus. The earlier cost-effectiveness models for influenza vaccine or varicella-zoster vaccine did not consider NPIs in an elaborate manner. Many challenges exist with conducting an economic evaluation involving COVID-19 response strategies especially with the dearth of vaccine data. At the same time, such evaluations may not be feasible upon the release of vaccine candidates, as prompt decisions by policy makers may be required within a limited time frame. Hence, this paper will also serve to guide the timely collection of key data fields before the arrival of vaccines, especially in settings where expertise in economic evaluation is limited.

For peer review only

## METHODS AND ANALYSIS

This paper describes the protocol of an economic evaluation of COVID-19 response measures with examples from the Singapore healthcare sector and societal perspective. The healthcare sector perspective, recommended by the Agency for Care Effectiveness (ACE) in Singapore,[5] is necessary to evaluate the impact of managing COVID-19, especially in circumstances where there are capacity limitations. The societal perspective, although not recommended by ACE, has been recommended by the Second Panel on Cost-effectiveness in Health and Medicine,[6] and is essential for understanding the broader social and economic costs attributed to the management of COVID-19. The methods and reporting of the study results will conform to the Consolidated Health Economic Evaluation Reporting Standards (CHEERS).[7] The study will commence on 01 Oct 2021, with a proposed completion date of 30 Jun 2022.

### Cost-effectiveness analysis



For peer review only - http://bmjopen.bmj.com/site/about/guidelines.xhtml

A cost-effectiveness analysis (CEA) provides a framework for comparing the incremental cost and incremental benefits of alternative policy options. To do so, the costs and benefits associated with each policy option must be estimated separately. A Markov cohort model is commonly used in CEA to simulate data beyond the observed period and also to integrate data inputs from multiple sources.[8] However, a Markov cohort model will be less appropriate for an infectious disease where considerations of dynamic transmission is critical to ensure an accurate prediction of events. The indirect effects of averted infections are also not captured in Markov models, potentially undervaluing the effects of an intervention.[9] In the context of a vaccination program, this is significant as the risk of infection among both vaccinated and non-vaccinated susceptible people decreases with vaccination as the extent of virus circulating in the community is reduced.

Hence, in this paper, we propose to adopt a Susceptible-Exposed-Infectious-Recovered (SEIR) transmission model (to be elaborated in the next section) and to attach cost and quality-adjusted life years (QALYs) to the outputs from the SEIR model (number of infections by disease severity). The difference in costs between the reference case and the policy alternative is divided by the difference in QALYs between the reference case and the policy alternative to obtain an incremental cost effectiveness ratio (ICER). The ICER will then be interpreted according to the willingness-to-pay threshold of the jurisdiction of interest.

In Figure 1, we provide an illustration of the possible combinations of policy options that should be evaluated. This list is derived from discussions with infectious disease clinicians, policy makers, health technology assessment experts and COVID-19 disease modelers. As an example in the Singapore context, the use of test-trace-isolate (TTI), mask and hygiene, and treatments for COVID-19,[10] are central to all policy strategies as they are not expected to be removed in the presence of a COVID-19 vaccine, particularly when vaccine supply is limited and 100% efficacy of the vaccine cannot be assumed. However, this should be adapted to each jurisdiction's context. In the reference case, travel restriction and social are considered together with the three policies described above. In the presence of the vaccine, the extent of relaxation of policies can be explored. This can be the complete removal of travel restriction (option 4) or social distancing (option 3) or both (option 1). In addition, a partial relaxation of all policy options in the reference case can be explored in the presence of a vaccine (option 2).

The key vaccine characteristics to be considered in this study will be guided by the World Health Organization (WHO) Target Product Profile (TPP),[11] as well as emerging data on the Pfizer, Moderna and Oxford-Astra Zeneca vaccines.[12-14] This include the indication of use, contradiction, target population, efficacy, safety, contraindications, dose regime, duration of protection, route of administration and product stability. The vaccination distribution strategies to be considered is determined by vaccine availability and population size to be vaccinated. Given the global demand for COVID-19 vaccines, it is unlikely that sufficient supplies are available for an entire country for several months from the release of a commercially available vaccine. Furthermore, manufacturers may choose to supply vaccines only to those who are willing to pay high prices. Hence, varying levels of vaccine supply availability should be examined in scenario analyses (e.g. 100,000 units per month, 500,000 units per month and 1,000,000 units per month). In addition, varying vaccine efficacy and prioritization of who should receive the limited vaccines will be considered for each vaccine distribution strategy.

**Model description**

An age structured extended SEIR model will be programmed in R using a differential equation model to generate the number of infections in Singapore over time by age group and stratified by disease severity (asymptomatic, mild-moderate, severe, very severe). A schematic outline of the transmission model is depicted in Figure 2. The population will be split into two subpopulations: those residing in the foreign



For peer review only - http://bmjopen.bmj.com/site/about/guidelines.xhtml

worker dormitories (FWD) and those residing outside of FWD. This allows for different COVID-19 policies between the two subpopulations as implemented in Singapore (e.g. more aggressive testing policy in FWD) as well as to capture the very different transmission in the two groups. Each subpopulation is further stratified into 16 age groups of 5 years interval starting from age 0. This is to account for varying probabilities of getting infected and developing symptoms at different severity levels and vaccine effectiveness according to age groups. The effectiveness of vaccines will be modelled based on the following parameters with vaccine reducing the 1) probability of getting infected when in contact with an infectious individual; 2) probability of being symptomatic after being infected; 3) probability of progression to severe disease post infection; 4) Infectiousness of infected individuals.

The interaction between the 32 groups (16 in FWD and 16 outside FWD) is described by contact matrices. For example, a subpopulation with 16 age groups at a location of interest (school/ workplace/ community) will generate a $16 \times 32$ contact matrix. The matrix will describe the average number of contacts with others made by an individual in a location of interest. The overall contact matrix for a subpopulation is a linear combination of the three location-specific contact matrices, with the coefficient for each location-specific matrix ranging between 0 to 1. The implementation of NPIs, which reduces interaction between individuals could be modelled by adjusting the relevant contact matrix coefficient (e.g. school closure modelled as a drop in the school contact matrix coefficient).

With the contact matrices and the probabilities of getting infected and developing symptoms, we could estimate the transmission rate for each age group in each subpopulation, and in turn estimate the force of infection at each time. For parameters for which there is great uncertainty or that influence the outcomes greatly, we will conduct sensitivity analyses, and assess how these results impact the conclusions reached. The entire population of Singapore based on population norms in 2019 will be simulated over a period of 1 year.

**Healthcare utilization**

We assume that for the general population there is no active surveillance although there are pockets of individuals such as those in high risk occupations who may need to undergo active surveillance. In addition, we assume that patients with asymptomatic infections do not incur any healthcare resources until they develop symptoms. In Singapore, all symptomatic patients will be isolated within the hospital. Patients with mild to moderate symptoms will be managed in isolation wards. Patients with severe symptoms will require ventilator but not intensive care. Patients with very severe symptoms will require intensive care.

**COVID-19 response policies (pharmaceutical and NPI) and its impacts**

The COVID-19 response policies included in this study are detailed in Table 1 and their impacts will be simulated in the transmission model. Briefly, the policies include: 1) TTI; 2) imposing travel restriction (international and/or domestic travel); 3) compulsory mask and improved hygiene practices; 4) social distancing: including workplace closures, school closures and work from home policies; 5) Dexamethasone and/or remdesivir use in COVID-19 patients with severe and very severe symptoms; 6) COVID-19 vaccination. A systematic review will be conducted to obtain efficacy estimates for the policies against COVID-19. The estimated impact of each policy, in the form of reduction in contacts, COVID-19 case numbers, or disease severity, will be utilized for this study. Data from the Singapore context will be used as much as possible, failing which, data sources from developed Asian economies such as Hong Kong, Korea and Japan are considered. The efficacy and safety data from the latest COVID-19 vaccine trials will be used, [12-14] and supplemented with the WHO TPP.[11] The most relevant control policies at the time of study completion will be reported.



For peer review only - http://bmjopen.bmj.com/site/about/guidelines.xhtml

**Table 1 Key COVID-19 response policies and descriptions**

| Policies | Sub-policies | Descriptions |
|---|---|---|
| Test-trace-isolate policy | Community testing | A combination of active testing within the community to identify positive cases, establish the chain of transmission and isolate positive cases to prevent further spread. |
| | Isolation of positive cases (or suspected positive cases) | Policy to require mild/moderate positive patients to be isolated either in their homes or in care facilities. This includes policy requiring international travelers to observe self-isolation for a fixed period of time. Where extensive contact tracing is available, people who have come into close contacts with COVID-19 positive patients are also required to serve stay-at-home notice or quarantine orders. |
| | Contact tracing initiatives | Government or state's initiatives to identify close contacts of COVID-19 cases to prevent the spread. |
| Travel restriction | - | International travel restriction implemented by the government to ban, limit or restrict international travelers. |
| Compulsory face masks and improved hygiene practices | - | Requirement to wear face masks or facial coverings when in public spaces. |
| Social Distancing | As-needed workplaces and schools closures | Requirement to shut down workplaces or educational facilities when a cluster or a number of cases are detected, in order to test exposed individuals and disinfect the affected locations. |
| | Compulsory work-from-home (WFH) policies | This policy is complementary with social lockdown. Companies are to continue its operation with their staff working from home. This policy may remain in place even after social lockdown has been lifted. |
| | Physical distancing | Sets of policies requiring social gathering to maintain minimum distance (1 to 2 meters) between members when outside of their homes or residences. This policy is often accompanied by restricting service capacity of business, mainly retails, malls and personal services to prevent overcrowding. |



For peer review only - http://bmjopen.bmj.com/site/about/guidelines.xhtml

| | | |
|---|---|---|
| Dexamethasone therapy | - | Intravenous dexamethasone 6mg once daily for up to 10 days among patients with severe and very severe symptoms |
| Remdesivir therapy | - | Intravenous remdesivir 200mg on day 1, followed by 100mg once daily for up to 10 days among patients with severe and very severe symptoms |
| Vaccination | - | Vaccination against SARS-CoV-2 |

**Measurement and valuation of health benefits**

Health benefits will be measured using quality-adjusted life years (QALYs). This is derived by multiplying the number of life years lived with a health utility weight to adjust for health-related quality of life (HRQoL). Published health utilities among patients with COVID-19 will be used.[15-17] When data on impact of long COVID on HRQoL emerge,[18] these may also be incorporated into the model. As medical isolation has shown to have negative impacts on health-related quality of life due to depression and anxiety, we will apply a reduction of QALY on all patients who were isolated for COVID-19.[19] In addition, we will estimate QALY losses due to vaccine-related adverse events based on influenza vaccine for mild/injection site reactions, severe anaphylaxis and severe grade 3 reactions requiring hospitalization.[20]

**Measurement and valuation of cost**

The summary of the sources of cost data and valuations are shown in Table 2. All costs will be expressed in 2021 Singapore dollars (SG$) as we expect that a vaccine will arrive only in 2021. This may be tweaked according to local circumstances as some countries have begun mass vaccination in December 2020. The analysis will be done for a time horizon of 1 year, assuming that the efficacy of the vaccine only lasts for one year. Hence no discounting will be applied for this study. Alternative time horizons and discount rates may be explored in scenario analyses.

**Table 2 Key study components and recommended sources**

| | | **Units** | **Remarks** | **Sources** |
|---|---|---|---|---|
| **Medical cost** | **COVID-19 treatment cost** | | | |
| | Mild/Moderate | $/Case | Estimated | Hospital database, Ministry of Health |
| | Severe | $/Case | Estimated | Hospital database, Ministry of Health |
| | Very severe | $/Case | Estimated | Hospital database, Ministry of Health |
| | Death | $/Case | Estimated | Hospital database, Ministry of Health |
| | **Vaccine related cost** | | | |



For peer review only - http://bmjopen.bmj.com/site/about/guidelines.xhtml

| | | | | |
|---|---|---|---|---|
| | Cost to treat adverse side effect (mild) | $/Case | Estimated | Literature review, COVID-19 vaccine trial data (when available) |
| | Cost to treat adverse side effect (severe) | $/Case | Estimated | Literature review, COVID-19 vaccine trials (when available) |
| | Cost per dose | $/Dose | Estimated | Government purchase agreements, hospital and clinic charges |
| | Cost of vaccine delivery device | $/Dose | Estimated | Government purchase agreements, hospital and clinic charges |
| **Productivity Loss** | **Number of productive days loss** | | | |
| | Mild/Moderate | Days | Based on length of hospital stay | Hospital database, Ministry of Health |
| | Severe | Days | Based on length of hospital stay | Hospital database, Ministry of Health |
| | Very severe | Days | Based on length of hospital stay | Hospital database, Ministry of Health |
| | Death | Days | Based on length of hospital stay | Hospital database, Ministry of Health |
| | **Population parameters** | | | |
| | Average age of infection/populatio n | Years | Calculated | Hospital database, Ministry of Health |
| | Age of retirement | Years | Estimated | Ministry of Manpower/Labour |
| | Expected life years | Years | Estimated | Worldometer, World Health Organization |
| | Employed Persons | Persons | Estimated | Ministry of Manpower/Labour, National Economic Survey |
| | Population of working adults (resident) | Persons | If applicable | Ministry of Manpower/Labour, National Economic Survey |



For peer review only - http://bmjopen.bmj.com/site/about/guidelines.xhtml

| | | | | |
|---|---|---|---|---|
| | Average monthly wage | $/Month | Calculated | Ministry of Manpower/Labour, National Economic Survey |
| | Average annual wage | $/Year | Calculated | Ministry of Manpower/Labour, National Economic Survey |
| | Interest rate | % | Estimated | Literature Review |
| | Discount rate | % | Estimated | Literature Review |
| | Wage loss per death | $/Death | Calculated based on average wage, average age, retirement age, discount rate | - |
| **Community testing** | Cost per swab test | $/Test | Estimated | Hospital database, Ministry of Health |
| **Contact tracing** | Average monthly wage of contact tracers | $/Month | Estimated | Ministry of Health, Ministry of Manpower |
| | Number of Close Contacts traced per case | Contacts/Case | Estimated | Ministry of Health, European Centre For Disease Control and Prevention guidance on contact tracing |
| | Number of Close Contacts traced per Officer | Contacts/Officer | Estimated | Ministry of Health, European Centre For Disease Control and Prevention guidance on contact tracing |
| **Community isolation/quarantine** | Number of close contact per positive case | Persons | Estimated | Ministry of Health, European Centre For Disease Control and Prevention guidance on contact tracing |
| | Numbers of quarantined days for close cases | Days | Estimated | Ministry of Health, European Centre For Disease Control and Prevention guidance on contact tracing |



For peer review only - http://bmjopen.bmj.com/site/about/guidelines.xhtml

| | | | | |
|---|---|---|---|---|
| **Travel restriction** | Accommodation receipts loss | $/Year | Estimated | External research databases, National Tourism survey, Economic surveys tourism sector |
| | Food & beverages receipt loss | $/Year | Estimated | External research databases, National Tourism survey, Economic surveys tourism sector |
| | Shopping receipt loss | $/Year | Estimated | External research databases, National Tourism survey, Economic surveys tourism sector |
| | Sightseeing, entertainment & gaming receipt loss | $/Year | Estimated | External research databases, National Tourism survey, Economic surveys tourism sector |
| | Others receipt loss | $/Year | Estimated | External research databases, National Tourism survey, Economic surveys tourism sector |
| | Monthly visitor | Visitor/Month | Estimated | External research databases, National Tourism survey, Economic surveys tourism sector |
| | Average visitor's expenditure | $/Visitor | Total tourism receipt / tourists | Based on tourism receipts and number of tourist arrivals: Total tourism receipt / Number of tourist arrival |
| **Mask Cost** | Cost per mask | $/Item | Estimated | Marketplaces price, B2B platform prices with mark up for distribution (if applicable) |



For peer review only - http://bmjopen.bmj.com/site/about/guidelines.xhtml

| | | | | |
|---|---|---|---|---|
| | Cost of masks distributed to the public per month | $/Month | Calculated based on cost of mask, population and distribution mark-up factor | - |
| **Social distancing** | Number of social distancing officers | Person | Estimated | Ministry of Health |
| | Average monthly wage of Social Distancing Officers | $/Month | Estimated | Ministry of Health, Ministry of Manpower |
| **Cost due to as-needed School Closure** | Probability of one case in school resulting in school cluster/school closure | Probability | Estimated | Ministry of Health, Ministry of Education |
| | Number of children per school | Children/School | Estimated | Ministry of Education |
| | Duration of school closure | Months | Estimated | Expert opinion |
| | Productivity loss of working dependents | % | Estimated | Literature review on productivity loss, Human resource reports and Business Management studies |
| **Cost due to as-needed workplace closure** | Probability of one case in workplace resulting in workplace cluster/workplace closure | Probability | Estimated | Ministry of Health |
| | Number of people affected per closure | People/Case | Estimated | Ministry of Health, Ministry of Manpower |
| | Duration of workplace closure | Months | Estimated | Ministry of Health |
| **Work from home policy** | Productivity loss from WFH | % | Estimated | Literature review on productivity loss, Human resource reports and Business Management studies |



For peer review only - http://bmjopen.bmj.com/site/about/guidelines.xhtml

| | | | | |
|---|---|---|---|---|
| | Proportion of workers working from home | % | Estimated | Ministry of Health, Ministry of Manpower |
| **Impact on retails and services (Limited social gathering)** | Loss in income from Retail and Other services | $/Month | Estimated | National economic survey |
| | Loss in income from retail sales (under full Circuit Breaker measures) | $/Month | Estimated | National economic survey |
| | Loss income from food & beverage (under full Circuit Breaker measures) | $/Month | Estimated | National economic survey |
| | Income loss after reopening | % | Estimated | National economic survey |
| **Economic Loss from unemployment** | Total unemployment increase | Cases/Month | Estimated | Based on national economic survey, reports, international databases |
| | Average duration of unemployment | Months | Estimated | Literature Review based on unemployment and transitioning |
| | Total wage loss due to unemployment resulted over 12-month period | $ | Unemployment x Wage x Unemployment duration | Based on average wage and duration of unemployment Unemployment x Wage x Unemployment duration x time horizon |
| **Vaccines parameters** | Number of doses required | Doses | Assumed | WHO Target Product Profile, COVID-19 vaccine trials (when available) |
| | Efficacy | % | Estimated | WHO Target Product Profile, COVID-19 vaccine trials (when available) |
| | Proportion of population covered by vaccine | % | Estimated | Ministry of Health, WHO subnational vaccine coverage data |



For peer review only - http://bmjopen.bmj.com/site/about/guidelines.xhtml

| | | | | |
|---|---|---|---|---|
| | Mild adverse reaction (injection reaction and systemic adverse reactions) | % | Estimated | WHO vaccine reaction rates, COVID-19 vaccine trials (when available) |
| | Severe anaphylaxis | % | Estimated | WHO vaccine reaction rates, COVID-19 vaccine trials (when available) |
| | Grade 3 adverse reactions requiring hospitalisation | % | Estimated | WHO vaccine reaction rates, COVID-19 vaccine trials (when available) |
| **Health-utility** | **COVID-19 related** | | | |
| | Healthy | QALY | Estimated | Literature review on COVID-19 health utilities |
| | Mild/moderate symptoms | QALY | Estimated | Literature review on COVID-19 health utilities |
| | Severe symptoms | QALY | Estimated | Literature review on COVID-19 health utilities |
| | Very severe symptoms | QALY | Estimated | Literature review on COVID-19 health utilities |
| | Death | QALY | Assumed as 0 | - |
| | **Medical isolation** | | | |
| | Disutility due to medical isolation | QALY | Estimated | Literature review on medical isolation |
| | **Vaccine-related side effects** | | | |
| | Mild adverse reaction (injection reaction and systemic adverse reactions) | QALY | Estimated | Literature review on influenza vaccination |
| | Severe anaphylaxis | QALY | Estimated | Literature review on influenza vaccination |



For peer review only - http://bmjopen.bmj.com/site/about/guidelines.xhtml

| | | | | |
|---|---|---|---|---|
| | Grade 3 adverse reactions requiring hospitalisation | QALY | Estimated | Literature review on influenza vaccination |

Based on the healthcare system's perspective, direct medical costs will include costs to manage the infections stratified by severity, cost to manage adverse events associated with the vaccine and costs of the vaccination programme. Costs to manage the infections include hospitalization, medications, laboratory tests, diagnostic tests, radiological examinations, use of ventilators, medical procedures, etc, where applicable. The cost of COVID vaccine will be based on the acquisition price, cost of accompanying delivery device (if any) and the costs associated with cold-chain management when it becomes available but may otherwise be estimated based on reported spending of UK and US government on COVID-19 vaccine deals.[21, 22]

From the societal perspective, direct non-medical costs such as childcare services and indirect cost such as lost productivity will be included. There are two possible approaches to quantifying the economic cost of NPIs. One approach, which is being explored by our team, is to use stock market performance as a proxy. This is based on the assumption that the stock market performance is sensitive to any announcement of NPIs, e.g. stock market index falls following announcement of border closure due to concerns over the impact on businesses. The change in stock market indices may then be tied to GDP to quantify the economic losses in absolute dollar terms. The reason for not using GDP directly is because GDP data are reported on a quarterly basis and may not be as sensitive to announcements of NPI compared to stock market indices. This approach of using stock market index as a proxy has been used in other studies to measure investors' sentiment and expected economic activities, which in turn reflect investors' own perceptions of the economic impacts of NPIs.[23, 24] Another approach is more labour intensive and is elaborated in the Supplementary appendix.

**Model calibration and validation**

Model calibration is necessary to ensure that model predictions are consistent with data sources informing the model inputs.[25] Hence, model parameter estimates will be calibrated with relevant empirical evidence. This can be done using the Bayesian approach, which involves defining probability distributions for model parameters, while considering evidence on the distribution of modelled outcomes and assumptions that relate model parameters and outcomes.[26] The study model will also be validated internally and externally: 1) face validity by clinical and modelling experts; 2) internal validation with checks for consistency, plausibility and debugging; 3) external validation with published literature.[27]

**Sensitivity analyses**

Sensitivity analyses is a critical component of a cost-effectiveness analysis to examine the large number of assumptions made as well as the uncertainties associated with model parameters. Threshold analysis may be performed to determine the threshold cost of the vaccine under the various target product profiles modelled. Scenario analysis will consider different time horizons, vaccine characteristics and vaccine prioritization policies in this study.

Value of information analysis (VIA) could be applied to measure the gains from additional research which reduce the uncertainty in the model parameters. In particular, based on the results from sensitivity analysis, we could identify which parameters the ICER is most sensitive to. Those parameters could be considered for additional research to reduce uncertainties. Then we can decide whether to



For peer review only - http://bmjopen.bmj.com/site/about/guidelines.xhtml

proceed with the additional research, by comparing the gains from the research (quantified in VIA) with the cost of conducting the research. If the additional research needed is prospective, VIA can also help to decide how much data to be further collected, thus optimizing the research design.

One limitation of using VIA here is that due to the urgency of COVID-19, new policies may need to be implemented immediately with the emergence of new clinical evidence. Hence there may not be sufficient time for additional prospective research before vaccine approval and distribution. However, VIA could help in at least two ways. Firstly, if VIA shows that reducing the uncertainty for selective parameters is of high value, it may be necessary to review if all existing information of those variables have been utilized. Secondly, VIA could inform the optimal post-licensure data collection needs for monitoring the benefit-risk profile of vaccines, which includes any possible enhancement impact in post-licensure studies, and will help to determine future vaccination strategies beyond the initial doses.

**Patient and public involvement**

Patients or the public will not be involved in the design, or conduct, or reporting, or dissemination plans of this research.

**DISCUSSION**

Several methodological challenges exist for conducting an economic evaluation of COVID-19 pandemic response strategies. Firstly, local data of the effectiveness of NPI may not be available for analysis. At the same time, effectiveness data of NPIs from another country setting are not directly applicable as they rely heavily on ecological factors, unlike pharmaceutical products that rely more on physiological and biological factors. Secondly, for vaccines, the long -term safety profile of COVID-19 vaccines will not be known, which may underestimate the risk associated with its use. In addition, uncertainties surrounding the long-term effectiveness of each vaccine candidate remains to be evaluated. Hence economic evaluation should be conducted once at the introduction of the vaccine with a subsequent analysis when more robust data is available. Thirdly, the population group to receive priority vaccination in the setting of limited vaccine supply remains to be determined. Available vaccine prioritization guidance by the WHO and Joint Committee on Vaccination and Immunisation have given health workers and the elderly the highest priority based on the goals of preserving health system capacity and minimizing COVID-related deaths.[28, 29] However, other jurisdictions may prioritize vaccination based on the goal of economic recovery, or a combination of both health-related and economic goals. These considerations would vary widely between jurisdictions, each with significant ethical considerations. Furthermore, other factors that may threaten vaccine uptake rates should be examined such as vaccine acceptance, payment models for vaccination, and expectations of daily living post-vaccination. Yet, efforts for economic evaluations of these COVID-19 pandemic response strategies should not be hampered as extensive sensitivity and scenario analysis of these known variables that contribute to large uncertainties can be conducted. Hence, a greater challenge would then lie in the interpretation of the results considering all these limitations.

As the COVID-19 pandemic continues, there are many trade-offs that need to be made among the various policy options. Economic evaluation can provide one source of input to inform this complex decision-making process. The protocol that we have described provides a detailed guide to estimate the broader economic costs of various NPIs that are being used, in line with other publications that have recommended the need to consider broader societal costs.[30, 31] Previous studies have so far established a cost inventory without specific guidelines or recommendations on how the costs should be estimated. Acemonglu et al presented optimal lockdown strategies in the US using trade-off between mortality and economy protection efforts without explicitly deriving the costs and associated



For peer review only - http://bmjopen.bmj.com/site/about/guidelines.xhtml

calculations from the relevant components.[32] Kim et al proposed a cost inventory of policy responses for COVID-19 and highlighted the importance of the considering broader societal costs without guidance on how to compute the relevant costs.[33]

The costing framework that we have developed aims to fill such gaps and to provide a standard tool through which economic evaluations for COVID-19 vaccines can be based upon. This framework also allows for the flexible analysis of different policy combinations according to a country's needs. Hence, the extent of relaxation of individual COVID-19 policies to be explored in the presence of a vaccine. By allocating specific cost associated with specific policies, this framework allows comparison between different policy combinations but more importantly to compare the costs between different scenarios. This is key to policy evaluation where long-term planning is highly time-sensitive and policies should be allowed to have flexible implementation. In addition, this protocol would also be of relevant to settings with limited economic evaluation expertise, as data management plans can be drawn up to facilitate timely collection of these essential variables to help accelerate the decision-making process.

In conclusion, we have described a costing framework which will be useful to researchers and policy makers who are interested to evaluate the cost-effectiveness of various policy options. The costing framework is described in great details to allow researchers and policy makers to add on relevant cost parameters which we have not considered, or remove cost parameters that are not relevant or are not available in their own jurisdictions.

**Author contributions:** HLW and HC conceived the study; HLW, HC and YT contributed to the design of the study and procurement of funding. BWBC, VAH, JL, FTG drafted the manuscript and all authors have read and approved the final manuscript.

**Funding:** The study was support by the National Medical Research Council, Ministry of Health, Singapore (COVID19RF3-0057). The National University of Singapore is a member of the International Decision Support Initiative, which facilitates funding support for publication fees from the Bill and Melinda Gates Foundation (OPP1202541). The National Medical Research Council, Ministry of Heath, Singapore and Bill and Melinda Gates Foundation have no direct role in the design of the study and collection, analysis, and interpretation of data and in writing the manuscript.

**Competing interests:** The authors declare they have no competing interests.

**Patient and public involvement:** Patients and/or the public were not involved in the design, or conduct, or reporting, or dissemination plans of this research.

**Patient consent for publication:** Not required.



For peer review only - http://bmjopen.bmj.com/site/about/guidelines.xhtml

## REFERENCES

1. Worldometer. COVID-19 Coronvirus Pandemic. https://www.worldometers.info/coronavirus/. Accessed 16 March 2021.
2. Asian Development Bank. COVID-19 Economic Impact Could Reach $8.8 Trillion Globally - New ADB Report. https://www.adb.org/news/covid-19-economic-impact-could-reach-8-8-trillion-globally-new-adb-report. Accessed 04 June 2020.
3. Council on Foreign relations. A Guide to Global COVID-19 Vaccine Efforts https://www.cfr.org/backgrounder/guide-global-covid-19-vaccine-efforts. Accessed 16 March 2021.
4. Decide Health Decision Hub. Model Fitness-for-Purpose Assessment Report. https://decidehealth.world/en/news/model-fitness-purpose-assessment-report. Accessed 09 September 2020.
5. Agency of Care Effectiveness. Medical technologies evaluation methods and process guide. https://www.ace-hta.gov.sg/docs/default-source/process-methods/ace-methods-and-process-guide-for-medical-technologies-evaluation-(1-oct-2018).pdf. Accessed 20 March 2021.
6. Sanders GD, Neumann PJ, Basu A, Brock DW, Feeny D, Krahn M, et al. Recommendations for conduct, methodological practices, and reporting of cost-effectiveness analyses: second panel on cost-effectiveness in health and medicine. JAMA. 2016;316:1093-103.
7. Husereau D, Drummond M, Petrou S, Carswell C, Moher D, Greenberg D, et al. Consolidated health economic evaluation reporting standards (CHEERS) statement. Int J Technol Assess Health Care. 2013;29:117-22.
8. Komorowski M, Raffa J. Markov models and cost effectiveness analysis: applications in medical research. Secondary analysis of electronic health records: Springer; 2016. p. 351-67.
9. Pitman R, Fisman D, Zaric GS, Postma M, Kretzschmar M, Edmunds J, et al. Dynamic transmission modeling: a report of the ISPOR-SMDM modeling good research practices task force-5. Value Health. 2012;15:828-34.
10. National Centre for Infectious Diseases. Interim treatment guidelines for COVID-19. https://www.ncid.sg/Documents/Interim%20Treatment%20Guidelines%20for%20COVID-19%20v4%20%2831%20Aug%202020%29-%20final.docx%20for%20upload.pdf. Accessed 16 December 2020.
11. World Health Organization. WHO Target Product Profiles for COVID-19 Vaccines. https://www.who.int/who-documents-detail/who-target-product-profiles-for-covid-19-vaccines. Accessed 04 June 2020.
12. Voysey M, Clemens SAC, Madhi SA, Weckx LY, Folegatti PM, Aley PK, et al. Safety and efficacy of the ChAdOx1 nCoV-19 vaccine (AZD1222) against SARS-CoV-2: an interim analysis of four randomised controlled trials in Brazil, South Africa, and the UK. Lancet. 2020.
13. Polack FP, Thomas SJ, Kitchin N, Absalon J, Gurtman A, Lockhart S, et al. Safety and efficacy of the BNT162b2 mRNA covid-19 vaccine. The New England journal of medicine. 2020.
14. Baden LR, El Sahly HM, Essink B, Kotloff K, Frey S, Novak R, et al. Efficacy and safety of the mRNA-1273 SARS-CoV-2 vaccine. The New England journal of medicine. 2021;384:403-16.
15. Ping W, Zheng J, Niu X, Guo C, Zhang J, Yang H, et al. Evaluation of health-related quality of life using EQ-5D in China during the COVID-19 pandemic. PloS one. 2020;15:e0234850.
16. Garrigues E, Janvier P, Kherabi Y, Le Bot A, Hamon A, Gouze H, et al. Post-discharge persistent symptoms and health-related quality of life after hospitalization for COVID-19. J Infect. 2020.
17. Arab-Zozani M, Hashemi F, Safari H, Yousefi M, Ameri H. Health-Related Quality of Life and its Associated Factors in COVID-19 Patients. Osong Public Health Res Perspect. 2020;11:296-302.
18. Meeting the challenge of long COVID. Nat Med. 2020;26:1803.



For peer review only - http://bmjopen.bmj.com/site/about/guidelines.xhtml

19. Sharma A, Pillai DR, Lu M, Doolan C, Leal J, Kim J, et al. Impact of isolation precautions on quality of life: a meta-analysis. J Hosp Infect. 2020;105:35-42.
20. Prosser LA, Bridges CB, Uyeki TM, Hinrichsen VL, Meltzer MI, Molinari NA, et al. Health benefits, risks, and cost-effectiveness of influenza vaccination of children. Emerg Infect Dis. 2006;12:1548-58.
21. The Straits Times: Britain nears $870 million Sanofi-GlaxoSmithKline Covid-19 vaccine deal: Report. https://www.straitstimes.com/world/europe/britain-nears-870-million-sanofi-glaxosmithkline-covid-19-vaccine-deal-report. Accessed 09 July 2020.
22. The Straits Times: In brief US secures 300 million possible vaccine doses. https://www.straitstimes.com/world/in-brief-us-secures-300-million-possible-vaccine-doses. Accessed 09 July 2020.
23. Narayan PK, Phan DHB, Liu G. COVID-19 lockdowns, stimulus packages, travel bans, and stock returns. Finance Res Lett. 2020:101732.
24. Ashraf BN. Economic impact of government interventions during the COVID-19 pandemic: International evidence from financial markets. J Behav Exp Finance. 2020;27:100371.
25. Vanni T, Karnon J, Madan J, White RG, Edmunds WJ, Foss AM, et al. Calibrating models in economic evaluation. Pharmacoeconomics. 2011;29:35-49.
26. Menzies NA, Soeteman DI, Pandya A, Kim JJ. Bayesian methods for calibrating health policy models: a tutorial. Pharmacoeconomics. 2017;35:613-24.
27. Eddy DM, Hollingworth W, Caro JJ, Tsevat J, McDonald KM, Wong JB. Model transparency and validation: a report of the ISPOR-SMDM Modeling Good Research Practices Task Force–7. Med Decis Making. 2012;32:733-43.
28. World Health Organization. WHO SAGE Roadmap For Prioritizing Uses Of COVID-19 Vaccines In The Context Of Limited Supply. https://www.who.int/publications/m/item/who-sage-roadmap-for-prioritizing-uses-of-covid-19-vaccines-in-the-context-of-limited-supply. Accessed 19 November 2020.
29. Joint Committee on Vaccination and Immunisation: updated interim advice on priority groups for COVID-19 vaccination. https://www.gov.uk/government/publications/priority-groups-for-coronavirus-covid-19-vaccination-advice-from-the-jcvi-25-september-2020/jcvi-updated-interim-advice-on-priority-groups-for-covid-19-vaccination. Accessed 19 November 2020.
30. Appleby J. Will covid-19 vaccines be cost effective—and does it matter? BMJ. 2020;371:m4491.
31. Neumann P, Cohen JT, Kim DD, Ollendorf DA. Consideration Of Value-Based Pricing For Treatments And Vaccines Is Important, Even In The COVID-19 Pandemic: Study reviews alternative pricing strategies (cost-recovery models, monetary prizes, advanced market commitments) for COVID-19 drugs, vaccines, and diagnostics. Health Aff. 2020:10.1377/hlthaff. 2020.01548.
32. Acemoglu D, Chernozhukov V, Werning I, Whinston MD. Optimal targeted lockdowns in a multi-group SIR model. NBER Working Paper. 2020;27102.
33. Daeho Kim D, Neumann PJ. Analyzing the Cost Effectiveness of Policy Responses for COVID-19: The Importance of Capturing Social Consequences. Med Decis Making. 2020;40:251-3.



For peer review only - http://bmjopen.bmj.com/site/about/guidelines.xhtml

**FIGURE LEGENDS**

Figure 1. Combination of COVID-19 pandemic response policies

Figure 2. SEIR transmission model schematics

For peer review only



For peer review only - http://bmjopen.bmj.com/site/about/guidelines.xhtml

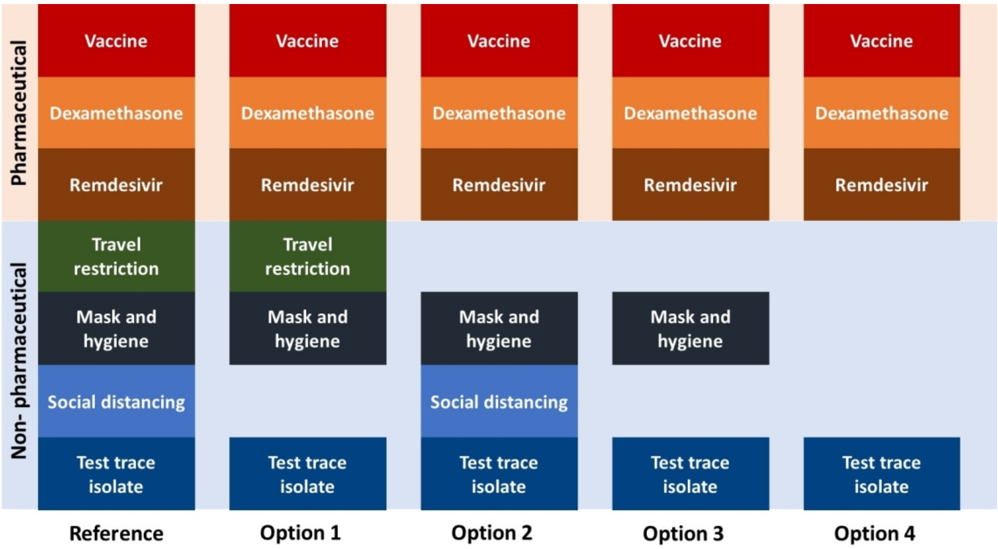


Combination of COVID-19 pandemic response policies

280x156mm (150 x 150 DPI)

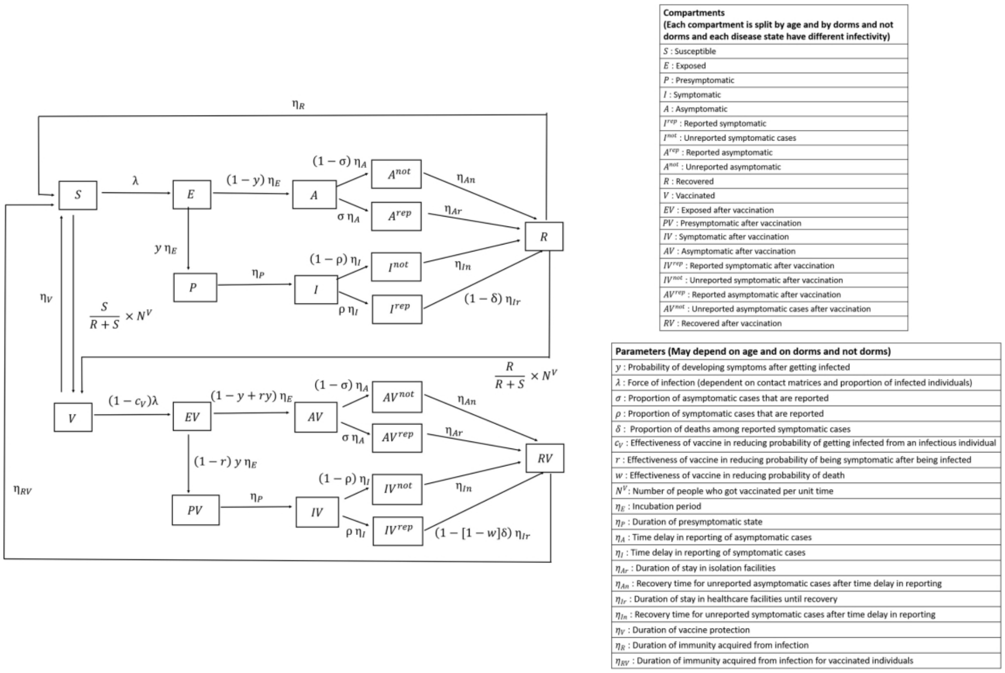


Susceptible Exposed Infectious Recovered transmission model schematics

424x283mm (144 x 144 DPI)

## SUPPLEMENTARY APPENDIX: QUANTIFYING ECONOMIC COST OF NON-PHARMACEUTICAL INTERVENTIONS

### Productivity loss

There are two types of productivity losses: COVID-19 infection related and wider population productivity loss. COVID-19 infection related productivity loss applies to those who are required to undergo isolation or quarantine because they are infected or are in close contacts to people with COVID-19 infection. The productivity loss for patients serving a compulsory quarantine order depends on the ability and productivity of working from home and the duration of the quarantine order. Wider population productivity loss occurs as a result of broad preventive NPIs such as workplace closure or workplace distancing as opposed to being directly affected by COVID-19. This productivity loss is attributable to the nature of jobs and services provided, level of infrastructure to support remote working, and individual companies' operations. Both categories of productivity loss must take into considerations the percentage of work that can be done from home. For example, Makridis et.al used digital-labour intensity for each industries in the US to estimate the proportion of work that can be done remotely.[1] Other countries such as Canada and Australia have made public servants work remotely even as the lockdown and social distancing eases.[2, 3] These policies for public servants can be used as a proxy to estimate the proportion of work that can be done remotely. Clearly, this is a variable that needs to be adapted to local settings.

In addition, productivity loss can be attributed to school closures. The United Nations Educational, Scientific and Cultural Organization has identified high economic costs among parents who miss work due to unavailable alternatives to childcare arrangements.[4] This could cause further strain the healthcare system if these parents are healthcare providers. Furthermore, there is large disparity in support for home learning between income levels.[5] This translate to loss of learning, especially among the disadvantaged minorities in the population. The United Nations has further identified the potential for rippled effects of school closures beyond learning with higher drop-out rates among poorer communities, which have direct economic consequences in potential future earnings for student affected.[6] This impact is particularly adverse during the foundation years of education, where a three-month school closure during grade 3 in developing countries is estimate to lead to higher drop-out rate by Grade 10,[7] and $16,000 income loss over a student's lifetime.[8]

Therefore, it is important to consider the opportunity costs due to productivity loss directly and indirectly attributable to COVID-19 and NPIs.

### Test-trace-isolate policies costs:

Contract tracing activities will depend on the number of positive cases identified. The European Centre For Disease Control and Prevention guidance on contact tracing has suggested resource estimates of 2-3 and 7-20 close contacts during lockdown and pre-lockdown period, respectively.[9] Contact tracing manpower operational cost depends on the efficiency of contact tracing networks (how may contacts each contact tracing staff can process) and the mobility of infected individuals (social contacts) multiplied by tracers' wages and the necessary infrastructure cost to maintain the operation.

### Travel restriction

To our knowledge, travel restriction has not been featured in any published cost-effectiveness analysis of vaccines given that border closures (between countries and between states/ provinces) of the current magnitude has been unprecedented. Global tourism and hospitality have experienced extreme decline.[10] Tourism and hospitality contribute a substantial portion to global economy in terms of

For peer review only

tourist receipts, employment, and infrastructure investments,[11] and is particularly critical for open economies such as Singapore. As such, border closure or travel restriction represents significant opportunity cost to countries. Opportunity cost of loss of tourism can be approximated from respective national economic databases. For example, in 2019, the Singapore tourism revenue reached SGD 27.7 billion (USD 20.4 billion), contributed 5.5% of the GDP and created 206,200 jobs.[12-14] In 2019, Vietnam tourism revenue reach USD 31 billion, contributed 6.3% of GDP, and created 4,951,000 jobs.[15] The difference of these revenue between 2020 and 2019 can be utilized as an estimation for the opportunity cost due to tourism.  This tourism revenue represents the opportunity cost of NPIs, such as the potential benefit gain when travel is resumed upon the introduction of COVID-19 vaccines. This is likely a conservative estimation of opportunity costs as tourism receipts are likely to have larger ripple effect throughout the economies due to multiplier effect, especially for those relying heavily on tourism sector such as Thailand and Vietnam.

**Compulsory face mask**

The implementation of compulsory face mask policy is currently not universal. Hence, this cost parameter may be dropped if a jurisdiction does not impose compulsory use of face mask. The costs should reflect the economic costs of the masks and the resources required for monitoring and enforcing the policy. First, mandatory face masks will incur a societal cost borne by either the citizens or by governments (in the case where masks are freely distributed to the population). Second, manpower and administrative cost are incurred in monitoring and enforcement of the policy. However, there needs to be careful consideration of the assignment of cost. For example, in Singapore, Safe Distancing Ambassadors (SDA) are employed by the government to ensure public compliance with masks and social distancing policies.[16] The SDAs are usually individuals who have been displaced from their current jobs. Hence, it will be appropriate to include the cost of hiring these individuals. However, in some countries, the monitoring and enforcement may involve redeploying existing manpower and such cost may not need to be considered. For example, UK redeploys its police force to ensure mask compliance.[17]

**Social distancing**

There are many disadvantages associated with prolonged school closures. Hence, it is very likely that as COVID-19 lingers on, most countries will adopt as-needed school closure in response to clusters forming in schools rather than indefinite school closure. As a result of school closures, parents and guardians (working dependents) of the affected students would incur productivity loss related to childcare activities such as time loss from work or direct non-medical cost such as hiring of babysitters and part-time domestic helpers to help with household chores. This can be estimated by approximating forced absenteeism among working caregivers due to school closures and wages forgone.[18] Studies have shown that 12-13 week school closure in the UK during influenza pandemic in 2009 led to 0.2-1% GDP reduction.

The same considerations may be applied to as-needed workplace closure. As the COVID-19 SEIR model incorporates contact matrices to characterise the spread of infections, it is possible to estimate the number of individuals who will be affected with each school or workplace closure.

Currently, the impacts of WFH policies or remote working settings on productivity are not well characterised. As such, to estimate the economic impacts of WFH, we will vary the productivity penalty. In our base case analysis, we will assume that there is a productivity loss of 10%. Current evidence from working from home (or remote working) based on a Chinese travel agency reported a 13% increase in

For peer review only

productivity.[19] However, in a HRM Asia report, 20% of the interviewed workers in Singapore reported decline in productivity and 33% reported an increase in productivity.[20]

We will estimate the proportion of the workforce that can work from home, which is up to 80% in Singapore,[21] which will differ in other jurisdictions, especially in lower-and-middle-income countries. This data can be obtained from published government guidelines for essential services, or using the percentage of workforce in each industry as a proxy. For example, employees with deskbound duties can work from home, while those in the manufacturing sector who require hands-on operations cannot.

Social distancing and/or partial lockdown have caused substantial economic loss to businesses. This loss can be estimated from economic data (retail sales, consumers expenditure, etc.) or projected based on weighted impacts from various sectors, ranging from hardest-hit contact-intensive personal care industries (up to 90% business loss) to mildly affected low-contact industries.[22, 23] For example, Leibovici et al. found that in the US, 85% of the production of high contact-intensive industries are directly consumed (final demand) as compared to 39% for low contact-intensive industries. This means a decline in high contact-intensive industries will lead to a greater rippled effect in consumption via input-output linkages. Furthermore, we believed there are rippled effects to the economy as consumers behaviours are quickly adapting to prolonged limited social gathering.[24]

According to IFO Institute for Economic Research (IFO), an estimate production loss ratio can be applied to various economic activities under strict lockdown scenarios.[25] We can apply this ratio and weighted composition of services sector to derive total economic loss. The robustness test can be done by varying the ratio to reflect specific country's lockdown stringency. For example, "motion pictures, video and television programme production" may be deemed as non-essential services and thus production level ratio can be set to 0 (instead of 1 in IFO's scenario) for strict social lockdown. In Singapore, using the lockdown production ratio provided in the IFO's calculation and sectoral GDP contribution in 2019 for Singapore, we expected that for a 4-week national lockdown (ie. "circuit breaker"), average lockdown production is 0.42, representing a 58% fall in production output in the month under lockdown.

In economies with heavy reliance on household consumptions for economic growth such as the US where final consumption of goods and services is at 82.1% of GDP in 2016,[26] social distancing measures that ban or restrict retail services will likely have major economic consequences.

For peer review only

**REFERENCES**


1. The Cost of COVID-19: A Rough Estimate of the 2020 US GDP Impact. https://www.mercatus.org/system/files/makridis-cost-covid-19-mercatus-v1.pdf. Accessed 23 July 2020.
2. Today: Canada public servants will still work remotely even as offices reopen – minister. https://www.todayonline.com/world/canada-public-servants-will-still-work-remotely-even-offices-reopen-minister. Accessed 23 July 2020.
3. The Guardian: Australian federal public servants finally told they can work from home during coronavirus crisis. https://www.theguardian.com/world/2020/mar/29/australian-federal-public-servants-finally-told-they-can-work-from-home-during-coronavirus-crisis. Accessed 23 July 2020.
4. United Nations Educational, Scientific and Cultural Organization: Adverse consequences of school closures. https://en.unesco.org/covid19/educationresponse/consequences. Accessed 22 November 2020.
5. Pew Research Centre: Lower-income parents most concerned about their children falling behind amid COVID-19 school closures. https://www.pewresearch.org/fact-tank/2020/04/15/lower-income-parents-most-concerned-about-their-children-falling-behind-amid-covid-19-school-closures/. Accessed 22 November 2020.
6. United Nations: Education during COVID-19 and beyond. https://www.un.org/development/desa/dspd/wp-content/uploads/sites/22/2020/08/sg_policy_brief_covid-19_and_education_august_2020.pdf. Accessed 22 November 2020.
7. RISE: Modeling the Long-Run Learning Impact of the COVID-19 Learning Shock: Actions to (More Than) Mitigate Loss. https://riseprogramme.org/publications/modeling-long-run-learning-impact-covid-19-learning-shock-actions-more-mitigate-loss. Accessed 22 November 2020.
8. The World Bank: COVID-19 Could Lead to Permanent Loss in Learning and Trillions of Dollars in Lost Earnings. https://www.worldbank.org/en/news/press-release/2020/06/18/covid-19-could-lead-to-permanent-loss-in-learning-and-trillions-of-dollars-in-lost-earnings. Accessed 22 November 2020.
9. European Centre for Disease Prevention and Control: Contact tracing for COVID-19: current evidence, options for scale-up and an assessment of resources needed. https://www.ecdc.europa.eu/sites/default/files/documents/COVID-19-Contract-tracing-scale-up.pdf. Accessed 07 July 2020.
10. Pew Research Center: More than nine-in-ten people worldwide live in countries with travel restrictions amid COVID-19. https://www.pewresearch.org/fact-tank/2020/04/01/more-than-nine-in-ten-people-worldwide-live-in-countries-with-travel-restrictions-amid-covid-19/. Accessed 15 July 2020.
11. TripAdvisor Strategic Insights: Sizing Worldwide Tourism Spending (or "GTP") & TripAdvisor's Economic Impact. https://mk0tainsightsjao4bom.kinstacdn.com/wp-content/uploads/2018/09/Worldwide-Tourism-Economics-2017-compressed.pdf. Accessed 19 July 2020.
12. Singapore Tourism Board: Tourism Sector Performance Q4 2019. https://www.stb.gov.sg/content/dam/stb/documents/statistics-marketing-insights/Quarterly-Tourism-Performance-Report/STB%20Q4%202019%20FA%20v7.pdf. Accessed 03 August 2020.
13. Department of Statistics: Singapore Economy 2019. https://www.singstat.gov.sg/modules/infographics/economy. Accessed 03 August 2020.
14. World Travel & Tourism Council, Singapore. https://tool.wttc.org/. Accessed 03 August 2020.
15. Forbes Vietnam: Vietnam welcomes 18 millions international tourists this year. https://forbesvietnam.com.vn/tin-cap-nhat/nam-nay-viet-nam-don-18-trieu-luot-khach-quoc-te-8674.html. Accessed 03 August 2020.


For peer review only

16. Today: Hiring of safe distancing ambassadors, enforcement officers to continue after circuit breaker ends: MEWR. https://www.todayonline.com/singapore/hiring-safe-distancing-ambassadors-enforcement-officers-continue-after-circuit-breaker. Accessed 01 July 2020.
17. The Telegraph: 'Unrealistic and unfair' to expect police to enforce face coverings in shops, says Police Federation. https://www.telegraph.co.uk/global-health/science-and-disease/coronavirus-news-face-masks-transport-compulsory-cases-deaths/. Accessed 23 July 2020.
18. Viner RM, Russell SJ, Croker H, Packer J, Ward J, Stansfield C, et al. School closure and management practices during coronavirus outbreaks including COVID-19: a rapid systematic review. Lancet Child Adolesc Health. 2020.
19. Bloom N, Liang J, Roberts J, Ying ZJ. Does working from home work? Evidence from a Chinese experiment. Q J Econ. 2015;130:165-218.
20. HRM Asia: A third of Singaporeans feel more productive working from home. https://hrmasia.com/a-third-of-singaporeans-feel-more-productive-working-from-home/. Accessed 02 July 2020.
21. The Straits Times: At least 350,000 still commuting to work in Singapore after reduction in essential services. https://www.straitstimes.com/singapore/at-least-350000-still-commuting-to-work-in-singapore-after-reduction-in-essential-services. Accessed 02 July 2020.
22. Federal Reserve Bank of St.Louis: How the Impact of Social Distancing Ripples through the Economy. https://www.stlouisfed.org/on-the-economy/2020/april/impact-social-distancing-ripples-economy. Accessed 23 July 2020.
23. Statistic Canada: Impact of social distancing measures on businesses, by business characteristics. https://www150.statcan.gc.ca/t1/tbl1/en/tv.action?pid=3310023701. Accessed 21 July 2020.
24. McKinsey & Company: Consumer sentiment and behavior continue to reflect the uncertainty of the COVID-19 crisis. https://www.mckinsey.com/business-functions/marketing-and-sales/our-insights/a-global-view-of-how-consumer-behavior-is-changing-amid-covid-19#. Accessed 22 July 2020.
25. Mandel A, Veetil VP. The economic cost of covid lockdowns: An out-of-equilibrium analysis. Available at SSRN 3588421. 2020.
26. The World Bank: Final consumption expenditure (% of GDP). https://data.worldbank.org/indicator/NE.CON.TOTL.ZS. Accessed 03 August 2020.

For peer review only
